# Evaluation of the enhanced upper limb therapy programme within the Robot-Assisted Training for the Upper Limb after Stroke trial: descriptive analysis of intervention fidelity, goal selection and goal achievement

**DOI:** 10.1177/0269215520953833

**Published:** 2020-09-11

**Authors:** Helen Bosomworth, Helen Rodgers, Lisa Shaw, Leanne Smith, Lydia Aird, Denise Howel, Nina Wilson, Natasha Alvarado, Sreeman Andole, David L Cohen, Jesse Dawson, Cristina Fernandez-Garcia, Tracy Finch, Gary A Ford, Richard Francis, Steven Hogg, Niall Hughes, Christopher I Price, Laura Ternent, Duncan L Turner, Luke Vale, Scott Wilkes, Hermano I Krebs, Frederike van Wijck

**Affiliations:** 1 Stroke Research Group, Population Health Sciences Institute, Newcastle University, Newcastle upon Tyne, UK; 2 Stroke Northumbria, Northumbria Healthcare NHS Foundation Trust, North Tyneside, UK; 3 Newcastle Hospitals NHS Foundation Trust, Newcastle upon Tyne, UK; 4 Population Health Sciences Institute, Newcastle University, Newcastle upon Tyne, UK; 5 School of Healthcare, University of Leeds, Leeds, UK; 6 Barking, Havering and Redbridge University Hospitals NHS Trust, Romford, UK; 7 London North West Healthcare NHS Trust, Northwick Park, UK; 8 Institute of Cardiovascular and Medical Sciences, University of Glasgow, Glasgow, UK; 9 Nursing, Midwifery & Health, Northumbria University, Newcastle upon Tyne, UK; 10 Medical Sciences Division, University of Oxford, and Oxford University Hospitals NHS Foundation Trust, Oxford, UK; 11 Lay investigator (contact Stroke Research Group, Population Health Sciences Institute, Newcastle University, Newcastle upon Tyne, UK; 12 NHS Greater Glasgow and Clyde, Glasgow, UK; 13 School of Health, Sport and Bioscience, University of East London, London, UK; 14 School of Medicine, University of Sunderland, Sunderland, UK; 15 Massachusetts Institute of Technology, Cambridge, USA; 16 School of Health and Life Sciences, Glasgow Caledonian University, Glasgow, UK

**Keywords:** Stroke, upper limb, rehabilitation, repetitive functional task practice, goals, fidelity

## Abstract

**Objective:**

To report the fidelity of the enhanced upper limb therapy programme within the Robot-Assisted Training for the Upper Limb after stroke (RATULS) randomized controlled trial, the types of goals selected and the proportion of goals achieved.

**Design:**

Descriptive analysis of data on fidelity, goal selection and achievement from an intervention group within a randomized controlled trial.

**Setting:**

Out-patient stroke rehabilitation within four UK NHS centres.

**Subjects:**

259 participants with moderate-severe upper limb activity limitation (Action Research Arm Test 0–39) between one week and five years post first stroke.

**Intervention:**

The enhanced upper limb therapy programme aimed to provide 36 one-hour sessions, including 45 minutes of face-to-face therapy focusing on personal goals, over 12 weeks.

**Results:**

7877/9324 (84%) sessions were attended; a median of 34 [IQR 29–36] per participant. A median of 127 [IQR 70–190] repetitions were achieved per participant per session attended. Based upon the Canadian Occupational Performance Measure, goal categories were: self-care 1449/2664 (54%); productivity 374/2664 (14%); leisure 180/2664 (7%) and ‘other’ 661/2664 (25%). For the 2051/2664 goals for which data were available, 1287 (51%) were achieved, ranging between 27% by participants more than 12 months post stroke with baseline Action Research Arm Test scores 0–7, and 88% by those less than three months after stroke with scores 8–19.

**Conclusions:**

Intervention fidelity was high. Goals relating to self-care were most commonly selected. The proportion of goals achieved varied, depending on time post stroke and baseline arm activity limitation.

## Introduction

Up to 80% of stroke survivors have difficulties using their affected arm in daily activities,^1^ which often persist in the longer term, impacting on the ability to engage social roles and on autonomy.^2^ There is a need for further high quality evidence to support interventions to improve arm function after stroke.^1,3,4^ Repetitive functional task training has shown promise for improving arm function,^3,5^ and therefore further trials of this type of intervention are particularly important. The Robot-Assisted Training for the Upper Limb after Stroke (RATULS) randomized controlled trial, the largest of its kind to date (*n* = 770), was published recently.^6^ Participants were randomized to receive robot-assisted training, an enhanced upper limb therapy programme (where repetitive functional task practice focused on personal goals), or usual care.^6^ There was little evidence of a difference in the primary outcome of arm activity limitation (i.e. success in attaining pre-specified improvement in the Action Research Arm Test^7,8^ score at three months) between randomization groups. However, participants who were randomized to receive the enhanced upper limb therapy programme performed significantly better in a number of secondary outcomes when compared to those who received usual care. Clinically important benefits at the end of the three month intervention period were observed in measures of impairment (Fugl-Meyer Assessment Motor Score),^8,9^ activities of daily living and mobility (Stroke Impact Scale).^10^ Additionally, there were statistically significant improvements which were not considered clinically important, as the confidence intervals did not include values that are currently deemed to be Minimum Clinically Important Differences. These statistically significant improvements were in measures of arm function (Action Research Arm Test), hand function (Stroke Impact Scale),^10^ and activities of daily living (Barthel Activity of Daily Living Index)^11^ – with the latter continuing to 6 months follow-up. Participants randomized to receive the enhanced upper limb therapy programme also performed significantly better than those randomized to receive robot-assisted training in measures of activities of daily living at three months (Stroke Impact Scale^10^ and Barthel Index^11^) but these improvements also did not reach the threshold for being considered clinically important.^6^

It is important that the development and fidelity of interventions are fully reported to enable the results of a trial to be interpreted, and for the intervention to be replicable in routine clinical practice or future research. However, stroke rehabilitation trials often fall short in terms of reporting these aspects.^12,13^ The development and description of the enhanced upper limb therapy programme followed the Template for Intervention Description and Replication (TIDieR) framework,^12^ and the planned delivery of the intervention (TIDieR items 1–11) has been reported.^14^ The aim of this paper is to report the intervention fidelity (TIDieR item 12) and a descriptive analysis of the types of personal goals selected and the proportion achieved.

## Methods

### Participants

Participants of the enhanced upper limb therapy programme were adults (age ⩾ 18 years) who were within one week and five years of their first stroke, for whom the stroke had resulted in moderate to severe upper limb activity limitation (Action Research Arm Test score 0–39 out of a maximum of 57).^7,8^

### Description of the enhanced upper limb therapy programme

The enhanced upper limb therapy programme comprised progressive, repetitive functional task practice focusing on participants’ personal goals, based on the concept of neuroplasticity,^15^ principles of skill acquisition,^16^ relevant evidence syntheses^1,3,5,17^ and our previous trials.^18–21^ Goal setting and monitoring goal achievement are known to increase motivation and engagement in therapy,^22^ whilst repetitive practice can improve arm activity limitation after stroke.^3,5^ A recent systematic review found that repetitive practice led to small but significant improvements in strength and activity of the affected upper limb.^23^ Furthermore, meta-analyses also reported significant improvements in arm activity limitation following at least 20 additional hours of repetitive practice,^3,5^ but as information about the actual number of repetitions is often poorly reported, uncertainty about the optimum amount remains.^24,25^

The aim of the enhanced upper limb therapy programme was to enable participants to achieve their personal goals by engaging their stroke-affected arm in functional activities, using appropriate everyday objects, linked to their goals. The enhanced upper limb therapy programme was designed to be meaningful, engaging and challenging, yet achievable.

**Figure 1.**
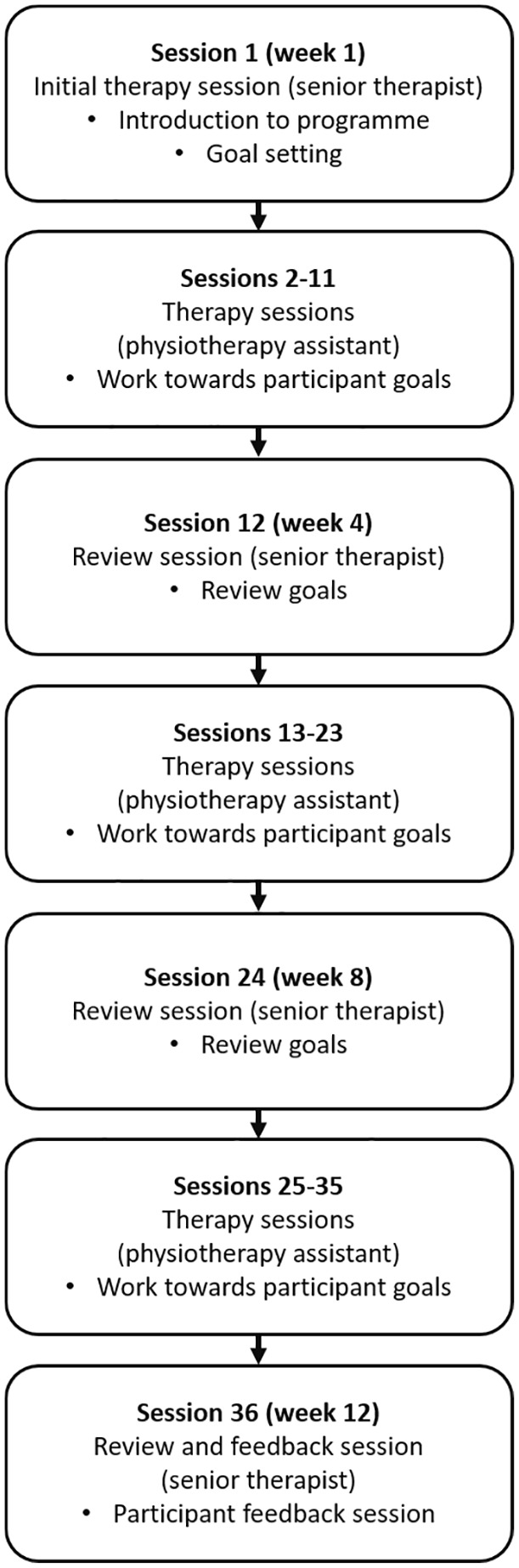
The enhanced upper limb therapy programme design.

This programme aimed to provide therapy sessions three times per week for 12 weeks (36 sessions total). Sessions were up to one hour, which included 45 minutes of face-to-face therapy for each participant (target 27 hours in total). An overview of the design of the enhanced upper limb therapy programme is shown in Figure 1. A senior therapist (physiotherapist or occupational therapist) undertook the initial therapy session and reviewed the participant every four weeks. Other therapy sessions were delivered by a physiotherapy assistant. At the start of their first session, participants were invited to identify personally relevant goals, which were not pre-specified other than that they should comprise a functional task involving the affected arm. Participants were advised, based on previous work, to select no more than four goals at each review session (up to 12 across the enhanced upper limb therapy programme).^26^ The goal selection process was not formalised but undertaken according to the senior therapist’s clinical judgement. In each session, participants practised functional activities to work towards their goals. Activities could be whole or part task practice.^27^ Part task practice was undertaken when a participant had difficulty with a specific part of a task, as it enabled them to concentrate on this particular aspect while working towards completing the task as a whole. The order in which activities were practised, the time spent on each, and the rate of progression were at the discretion of the senior therapist or physiotherapy assistant and participant. Participants were encouraged to undertake as many repetitions as possible within each session. There was no set target for the number of repetitions.

At therapy session 12 (end of week 4) and 24 (end of week 8), progress towards goals was reviewed. If a participant had achieved a goal, a new goal was selected. If a participant found a goal or activity too challenging or experienced other problems, an alternative goal and activity were chosen. At the final therapy session, practice continued and progress towards goals was reviewed with a senior therapist. Part of this session was also dedicated to providing feedback to the participant about progress over the course of the programme and advice about maintaining arm function in the longer term.

Transport was arranged for participants to attend all therapy and review sessions by the local study co-ordinator if required.

Training was provided to the senior therapists and physiotherapy assistants who delivered the enhanced upper limb therapy programme, with updates and ongoing training throughout the trial. In addition, three manuals provided guidance on intervention delivery (see supplementary material). The first described the purpose, principles and structure of the enhanced upper limb therapy programme as well as staff roles and responsibilities, the second provided guidance on how to structure each session (including assessment, ‘warm up’ and stretching, demonstration and education, progression, monitoring compensatory movements and feedback). The third manual provided examples of soft tissue stretches prior to task practice, and an overview of commonly selected goals. The most commonly selected goals from our previous arm rehabilitation studies were prepared as examples to facilitate goal selection^18–21^ (i.e. washing, dressing, eating and drinking activities), with accompanying step-by-step flowcharts to guide and progress practice of functional tasks, each with whole-task and part-task options.

### Fidelity of the enhanced upper limb therapy programme

‘Fidelity of implementation refers to the degree to which . . . providers implement programs as intended by the program developers’,^28^ and includes adherence (e.g. content, dose) whilst moderators (e.g., intervention complexity, facilitation strategies such as training and manuals) may affect fidelity.^29^ This paper reports the fidelity of the enhanced upper limb therapy programme which includes adherence to the intervention as it was intended to be delivered – but does not include analysis of moderators.

A bespoke proforma was completed by the trial senior therapists and physiotherapy assistants for each participant to document the content of their enhanced upper limb therapy programme (see supplementary material). The following were recorded: session attendance; duration of session; duration of face-to-face therapy within a session; number of repetitions per task practised in each session; goals selected and type of task practice (whole or part task); and goals achieved.

In terms of repetitions, for whole task practice, completion of the whole task counted as one repetition, that is, from the start position to a ‘return to the start position’ or to completion of the task (if different from the start position). For part task practice, completion of the component of the part task counted as one repetition.

Details about the goals selected were recorded at the initial therapy session and at the four and eight week review sessions. Information about whether a participant had achieved their goals (recorded as ‘Yes’/ ‘No’ according to the senior therapist’s clinical judgement) was collected at the four, eight and 12 week review sessions. A formal goal attainment scale was not used.

To monitor intervention provision, summary data about the enhanced upper limb therapy programme were reviewed and reports about fidelity were sent to study centres every six months. This was followed by a discussion with the coordinating centre team about their performance.

### Data analysis

Information about: session attendance; duration of session; duration of face-to-face therapy within each session; number of repetitions per session, number of goals selected; type of task practice (whole or part task); and number of goals achieved were analysed descriptively. Analyses of numeric data from are presented as mean (SD) or median [IQR] as appropriate. Categorical data are presented as *n* (%).

Recorded goal descriptions were reviewed by a research physiotherapist and retrospectively coded into categories based upon the Canadian Occupational Performance Measure^30^: self-care, productivity and leisure, and their respective sub-categories. An additional ‘other’ category was developed where the described goal did not fit into one of the three Canadian Occupational Performance Measure categories. The ‘other’ sub-categories were coded as: generic pick up/ grasp/ reach/ place object; range of movement; upper limb strengthening; and weight bearing. In addition, where there was insufficient information in the free-text field on the proforma to code a goal, this was coded as ‘unclassified’.

## Results

### Participants

Between the 14th April 2014 and 30th April 2018, 259 trial participants were randomized to receive the enhanced upper limb therapy programme. The characteristics of participants are shown in Table 1. The median time from stroke was 258 days [IQR 115–546]. The level of arm activity limitation at baseline was severe: out of a maximum of 57, the median [IQR] Action Research Arm Test score was 3 [0–13].^6,31^

**Table 1.** Characteristics of participants randomized to receive the enhanced upper limb therapy programme.

Sex: *n* (%)	*n* = 259
Male	159 (61.4%)
Female	100 (38.6%)
Age at randomization (years)	*n* = 259
Mean (SD)	59·4 (14.3)
Time from stroke to randomization (days): *n* (%)	*n* = 259
<3 months	46 (17.8%)
3 to 12 months	117 (45.2%)
>12 months	96 (37.1%)
Stroke type: *n* (%)	*n* = 259
Cerebral infarction	202 (78.0%)
Primary intracerebral haemorrhage	56 (21.6%)
Subarachnoid haemorrhage	1 (0.4%)
National Institute of Health (NIH) Stroke Scale total score	*n* = 259
Mean (SD)	5·7 (3.2)
Arm affected by the stroke: *n* (%)	*n* = 259
Right	116 (44.8%)
Left	143 (55.2%)
Handedness: *n* (%)	*n* = 259
Right	223 (86.1%)
Left	35 (13.5%)
Ambidextrous	1 (0.4%)

In line with the RATULS trial, the categories of baseline Action Research Arm Test score 0 to 7, 8 to 19 and 20 to 39 were used to characterize severity of upper limb functional limitation.^31^ A total of 175/259 (68%) participants had severe upper limb activity limitation (Action Research Arm Test 0–7), 31/259 (12%) had a baseline Action Research Arm Test score of 8 to 19, and 53/259 (20%) had a baseline Action Research Arm Test score of 20 to 39.

For time since stroke, pre-defined categories of <3 months, 3 to 12 months and >12 months were used.^31^ A total of 46/259 (18%) of participants were less than 3 months post stroke at randomization, 117/259 (45%) were 3 to 12 months post stroke and 96/259 (37%) were more than 12 months post stroke.

### Intervention fidelity

Table 2 summarizes the intervention fidelity data. Overall, 84% of all possible sessions were attended. The most common reason for lack of attendance at sessions was that the participant was unwell (406/1447 (28%) sessions which were not attended). Further details about lack of attendance have been reported previously.^6^

**Table 2.** Fidelity of the enhanced upper limb therapy programme.

	Target	Actual	
Overall number of sessions attended	9324	*n* (%)	7877 (84%)
Total number of sessions attended per participant	36	Median [IQR]	34 [29–36]
Overall number of review sessions attended	1036	*n* (%)	941 (91%)
Total number of review sessions attended per participant	4	Median [IQR]	4 [4–4]
Total duration of therapy sessions (hours:minutes) per participant	36 hours	Median [IQR]	30:32 [24:42–34:05]
Duration of each therapy session (minutes) per participant	60 minutes	Median [IQR]	60 [45–60]
Total duration of face-to-face therapy within sessions (hours:minutes) per participant	27 hours	Median [IQR]	24:40 [20:24–26:15]
Duration of face-to-face therapy within each session (minutes) per participant	45 minutes	Median [IQR]	45 [45–45]

In terms of overall duration of therapy sessions, the average duration of therapy sessions was 85% of the target of 36 hours. Within the therapy sessions, 91% of the target of 27 hours of face-to-face therapy was achieved.

There was no target for the number of repetitions to be achieved by participants. A median of 127 [IQR 70–190] repetitions were achieved per participant per session attended. Overall, a median of 4121 [IQR 2395–5727] repetitions per participant were achieved across the enhanced upper limb therapy programme. Participants predominantly used whole task practice to work towards their goals; 2213/2664 (83%) of repetitions were whole task whilst 451/2664 (17%) were part task. Table 5 presents the number of repetitions undertaken, categorized according to baseline Action Research Arm Test score and time since stroke. These findings indicate that the highest median number of repetitions was undertaken by participants with Action Research Arm Test scores between 0 and 7 who were less than three months post stroke, whilst the lowest median number of repetitions was undertaken by those who were more than 12 months post stroke, with Action Research Arm Test scores between 20 and 39. There was considerable variation in the number of repetitions completed as indicated by the large interquartile range across all groups, whilst the interquartile ranges also overlapped considerably.

### Goals selected and achieved

Table 3 shows the Canadian Occupational Perfor-mance Measure categories and the subcategories of chosen goals. The most popular category of goal choice was ‘self-care’ (54%), followed by the ‘other’ category (25%), ‘productivity’ (14%) and ‘leisure’ (7%). In the ‘self-care’ category, the subcategory of ‘personal care’ was the most frequently selected, comprising 88% of goals. Of the 1449 self-care goals, the majority (622/1449 (43%)) were related to eating and drinking, whilst 254/1449 (18%) were related to dressing. All 374 goals in the productivity category related to household management, with 206/374 (55%) of goals being in the category of ‘cleaning’ and 125/374 (33%) goals in the category ‘cooking’. In the ‘leisure’ category the most popular subcategory was ‘correspondence’ (92/180 (51%)). The ‘other’ category predominantly comprised of impairment-based upper limb goals with ‘range of movement’ being the most commonly chosen goal in that category 413/661 (62%). Range of movement was the second most commonly chosen goal overall. A total of 163/661 (25%) goals within the ‘other’ category comprised of generic reach, grasp, pick-and place activities which were not specified sufficiently to be allocated to any particular Canadian Occupational Performance Measure subcategory. There was insufficient information to subcategorize 26/661 (4%) of ‘other’ goals.

**Table 3.** Canadian Occupational Performance Measure^25^ goal choice, activities and goal achievement.

Category	Number of goals in category (%)	Number of goals achieved in category (%)*	Subcategory	Number of goals in each subcategory (%)	Number of goals achieved in each subcategory (%)*	Activity category	Number of goals in each activity category (%)	Number of goals achieved in each activity category (%)*
Leisure	180/2664 (7%)	109/171 (64%)	Active recreation	16/180 (9%)	10/14 (71%)	Outings	0/ 16 (0 %)	0/ 0 (0%)
						Sports	16/16 (100%)	10/14 (71%)
						Travel	0/16 (0%)	0/0 (0%)
			Quiet recreation	71/180 (39%)	41/69 (59%)	Crafts	4/70 (6%)	3/4 (75%)
						Hobbies	63/70 (90%)	35/62 (56%)
						Reading	3/70 (4%)	3/3 (100%)
			Socialization	93/180 (52%)	58/ 88 (66%)	Correspondence	92/93 (99%)	57/87 (66%)
						Parties	0/93 (0%)	0/0 (0%)
						Phone calls	0/93 (0%)	0/0 (0%)
						Visiting	1/93 (1%)	1/1 (100%)
Productivity	374/2664 (14%)	211/351 (60%)	Household management	374/374 (100%)	211/351 (60%)	Cleaning	206/374 (55%)	100/189 (53%)
						Cooking	125/374 (33%)	81/121 (67%)
						DIY	11/374 (3%)	9/11 (82%)
						Laundry	19/374 (5%)	13/19 (68%)
						Using light switches	13/374 (3%)	8/11 (73%)
			Paid/unpaid work	0/374 (0%)	n/a	Finding/ keeping a job	0/0 (0%)	0/0 (0%)
						Volunteering	0/0 (0%)	0/0 (0%)
			Play/ school	0/374 (0%)	n/a	Homework	0/0 (0%)	0/0 (0%)
						Play skills	0/0 (0%)	0/0 (0%)
Self-care	1449/2664 (54%)	640/ 1354 (47%)	Community management	25/1449 (2%)	19/23 (83%)	Finances	19/25 (76%)	16/17 (94%)
						Shopping	0/25 (0%)	0/0 (0%)
						Transportation	6/25 (24%)	3/6 (5%)
			Functional mobility	141/1449 (10%)	65/136 (48%)	Indoor	42/141 (30%)	24/40 (60%)
						Outdoor	7/141 (5%)	4/7 (57%)
						Transfers	92/141 (65%)	37/89 (42%)
			Personal care	1283/1449 (89%)	556/1195 (47%)	Bathing	166/1283 (13%)	79/150 (53%)
						Childcare	4/1283 (<1%)	0/3 (0%)
						Dressing	254/1283 (20%)	0/1 (0%)
						Eating and drinking	622/1283 (48%)	234/587 (40%)
						Grooming	74/1283 (6%)	32/69 (46%)
						Hygiene	161/1283 (13%)	70/142 (49%)
						Medication	1/1283 (<1%)	0/1 (0%)
Other	661/2664 (25%)	327/625 (52%)	Pick up/grasp/reach/place object	163/661 (25%)	84/155 (54%)	n/a		
			Range of movement	413/661 (62%)	196/390 (50%)			
			Upper limb strengthening	8/661 (1%)	7/7 (100%)			
			Unclassified	26/661 (4%)	15/24 (63%)			
			Weight bearing	51/661 (8%)	25/49 (51%)			

An additional ‘other’ category was developed where the described goal did not fit into one of the three Canadian Occupational Performance Measure categories. *Data not available for 163/2664 goals.

A median of 12 goals [IQR 9–12] were selected per participant during the 12 week enhanced upper limb therapy programme. Of the 2664 goals selected, goal achievement data were recorded for 2501 (94%). In total, 1287/2501 (51%) of goals were achieved, ranging between 47% and 100% for each Canadian Occupational Performance Measure subcategory (Table 3). A median of 5 goals [IQR 2–7] were achieved per participant. Of the three most commonly chosen goals, 234/587 (40%) related to eating and drinking, 196/390 (50%) related to range of movement and 141/242 (58%) related to dressing were achieved.

To better understand who were most or least successful in achieving their personal goals with the enhanced upper limb therapy programme, the impact of baseline Action Research Arm Test score and time since stroke on goal selection and goal achievement was explored.

Goal achievement varied according to baseline Action Research Arm Test score and time since stroke (Table 4). Those participants who had the lowest baseline Action Research Arm Test score (0–7) and who were recruited more than one year after stroke had the lowest goal achievement with only 167/616 (27%) goals being achieved. Of the goals related to self-care (which includes personal care and functional mobility), only 23% were achieved in this group. In contrast, participants in all three baseline Action Research Arm Test score categories who were less than three months after stroke achieved between 73% and 88% of their goals.

**Table 4.** Goal choices and goal achievement according to baseline ARAT score and time since stroke.

Baseline ARAT (*n* = participants)	Time since stroke (*n* = participants)	Number of goals selected (%)	Number of goals achieved (%)*	Category	Number of goals selected in category (%)	Number of goals achieved in category (%)*
ARAT 0 to 7 (*n* = 175)	<3 months (*n* = 26)	262/1759 (15%)	175/240 (73%)	Leisure	18/262 (15%)	17/18 (76%)
				Productivity	22/262 (8%)	15/22 (68%)
				Self-care	118/262 (45%)	76/107 (71%)
				Other	104/262 (40%)	71/94 (76%)
	3 to 12 months (*n* = 79)	823/1759 (47%)	342/781 (44%)	Leisure	27/823 (3%)	11/26 (42%)
				Productivity	142/823 (17%)	75/133 (56%)
				Self-care	480/823 (58%)	178/454 (39%)
				Other	174/823 (21%)	78/168 (46%)
	>12 months (*n* = 70)	674/1759 (38%)	167/616 (27%)	Leisure	16/674 (2%)	2/14 (14%)
				Productivity	91/674 (17%)	29/80 (36%)
				Self-care	361/674 (54%)	77/332 (23%)
				Other	206/674 (31%)	59/190 (31%)
ARAT 8 to 19 (*n* = 31)	<3 months (*n* = 5)	48/333 (14%)	36/41 (88%)	Leisure	3/48 (6%)	3/3 (100%)
				Productivity	6/48 (13%)	4/5 (80%)
				Self-care	24/48 (50%)	16/19 (84%)
				Other	15/48 (31%)	13/14 (93%)
	3 to 12 months (*n* = 13)	136/333 (41%)	72/128 (56%)	Leisure	8/136 (6%)	3/7 (43%)
				Productivity	10/136 (7%)	8/10 (80%)
				Self-care	95/136 (70%)	46/89 (52%)
				Other	23/136 (17%)	15/22 (68%)
	>12 months (*n* = 13)	149/333 (45%)	93/149 (62%)	Leisure	10/149 (7%)	7/10 (70%)
				Productivity	19/149 (13%)	16/19 (84%)
				Self-care	78/149 (52%)	45/78 (58%)
				Other	42/149 (28%)	25/42 (60%)
ARAT 20 to 39 (*n* = 53)	<3 months (*n* = 15)	168/572 (29%)	124/162 (77%)	Leisure	29/168 (17%)	26/27 (96%)
				Productivity	26/168 (15%)	17/25 (68%)
				Self-care	77/168 (46%)	54/74 (73%)
				Other	36/168 (21%)	27/36 (75%)
	3 to 12 months (*n* = 25)	274/572 (48%)	202/267 (76%)	Leisure	61/274 (22%)	38/59 (64%)
				Productivity	48/274 (18%)	39/47 (83%)
				Self-care	134/274 (49%)	130/104 (80%)
				Other	31/274 (11%)	21/31 (68%)
	>12 months (*n* = 13)	130/572 (23%)	76/117 (65%)	Leisure	8/130 (6%)	6/8 (75%)
				Productivity	10/130 (8%)	8/10 (80%)
				Self-care	82/130 (63%)	44/71 (62%)
				Other	30/130 (23%)	18/28 (64%)

* Data not available for 163/2664 goals.

**Table 5.** The number of repetitions undertaken, categorized according to baseline Action Research Arm Test score and time since stroke.

Baseline ARAT (*n* = participants)	Time since stroke (*n* = participants)	Total number of repetitions achieved (Median [IQR])
ARAT 0 to 7 (*n* = 175)	<3 months (*n* = 26)	4682 [1703–5754]
	3 to 12 months (*n* = 79)	3855 [2395–5820]
	>12 months (*n* = 70)	3981 [2445–5273]
ARAT 8 to 19 (*n* = 31)	<3 months (*n* = 5)	3008 [1421–6303]
	3 to 12 months (*n* = 13)	4430 [3406–7594]
	>12 months (*n* = 13)	3455 [1897–6306]
ARAT 20 to 39 (*n* = 53)	<3 months (*n* = 15)	3710 [1783–5562]
	3 to 12 months (*n* = 25)	4663 [3565–6093]
	>12 months (*n* = 13)	3200 [882–6676]

## Discussion

We have described the fidelity, types of goals selected and proportion achieved, of a repetitive functional task practice intervention for stroke survivors with moderate to severe arm activity limitation, which was used in the NIHR HTA RATULS randomized controlled trial. The RATULS trial reported clinically important and statistically significant benefits in a number of secondary outcomes when compared with usual care (i.e. arm impairment, activities of daily living and mobility).^31^ To our knowledge, this is the first description of an intervention aimed at improving arm activity after stroke that comprises a detailed account of adherence (i.e. the number of sessions attended; session duration; duration of face-to-face therapy; goals selected and type of task practice (whole or part task); the number of repetitions of tasks and goals achieved), undertaken within a large multicentre randomized controlled trial of this type. Stroke rehabilitation trials often fall short in terms of reporting the details of the interventions provided, however the description of the enhanced upper limb therapy programme complies with the current recommendations.^12,13^

As treatment parameters were provided as described in the protocol and study manuals, fidelity to the enhanced upper limb therapy programme in the RATULS trial was high. This indicates that it is possible to standardize the delivery of a complex intervention of this nature, whilst allowing therapists to tailor the specific intervention parameters to the needs, goals and characteristics of individual participants – a prerequisite for person-centred rehabilitation.^32^ The high level of fidelity also confirms that it was possible for this complex intervention to be delivered by physiotherapy assistants, who were trained in the intervention delivery and supervised by experienced therapists. In addition to adherence, moderating factors are also thought to influence fidelity, including intervention complexity, facilitation strategies (e.g. training, therapy manuals), quality of delivery (e.g. monitoring and feedback for therapists) and participant responsiveness (i.e. their engagement in the intervention).^29^ Training and therapy manuals, as well as guidance and feedback were provided to therapists, but these factors were not formally analysed. However, the views of trial participants and healthcare professionals about the factors that affected the implementation of the trial were explored in a process evaluation that was conducted alongside the RATULS trial, and these findings have been reported elsewhere.^31^ In addition, participant responsiveness may have been influenced by fatigue, which is common post stroke.^33^ The RATULS process evaluation indicated that some participants found the intervention physically and cognitively tiring, but despite this, participants generally appeared motivated to further their recovery by engaging in this treatment.^31^

The three most commonly chosen goals selected by participants in this study were eating and drinking, improving range of movement and dressing, and this information may be helpful for clinicians working with stroke patients with moderate to severe arm activity limitation. Despite the specification that goals should comprise a functional task involving the affected the arm, improving range of movement was commonly chosen. This finding is likely to reflect the level of severe arm impairment in our study population and the difficulty of setting goals around functional activities for these participants. The overall proportion of goals achieved was low (51%), but further exploration showed that participants who were less than three months after stroke did achieve the majority of their goals. In contrast, many participants with severe activity limitation of the affected arm (Action Research Arm Test 0–7) who were more than three months post stroke, had difficulty achieving their goals, in particular those related to self care (including personal care and functional mobility). This may be explained by a number of potential factors in addition to paresis, including spasticity and contractures,^34^ as well as learned non-use,^35^ which may have become established in this subgroup. These factors, including other co-existing impairments (e.g. cognitive impairment) were assessed clinically as they informed the goal setting process, but were not formally measured as part of the trial, and therefore could not be included in the analysis and interpretation of the findings. Compared to a pilot study (3 sites; *n* = 55) that evaluated a similar enhanced upper limb therapy programme, the types of goals selected were similar but the overall proportion of goals achieved was much higher at 92%.^21^ However in that study, participants were within two weeks post stroke with a higher level of arm function and therefore had a more favourable prognosis.^36^ Most other repetitive task training studies do not report data on goal selection or achievement, so comparisons cannot be made. In the context of treatment for arm spasticity, a review of five studies found that 46% of goals related to symptoms or impairment and 54% related to activities, with between 27% and 72% of goals relating to active function and mobility being achieved.^37^

Achieving goals depends on a number of inter-related factors, including: the method for selecting goals and recording their achievement, as well as the appropriateness of therapeutic input (in terms of content and dose) in relation to the participant’s abilities and needs. Goal setting is currently considered to be integral to best practice in stroke rehabilitation and is recommended in national clinical guidelines.^32^ The evidence that this approach improves clinical outcomes is limited however, and there appears to be no consensus about the optimum approach.^38,39^ It was decided not to use a formal goal setting method (e.g. the Canadian Occupational Performance Measure,^30^ or the Goal setting and Action Planning framework^40^) in the RATULS trial, as we endeavoured to reflect clinical practice in the UK.^41^ It was challenging to set meaningful, functional goals that are achievable with stroke patients with severe arm activity limitation. The prognosis for those with severe arm impairment who are three months or longer after stroke, when spontaneous recovery tends to slow down,^36^ is generally unfavourable. These participants were included in the RATULS trial as there was evidence that they might benefit from the biomechanical advantage of robot-assisted training,^42^ which enabled participants to engage in repetitive practice, whilst having the weight of their affected arm supported by the device. Some patients with severe impairment and little chance of upper limb recovery may select aspirational rather than achievable goals, which may enhance motivation – but may also lead to unrealistic expectations, and difficulty adjusting to the residual consequences of stroke. A key component of goal setting is managing expectations, but the way in which goal setting was undertaken in this study was not documented, which was a limitation. Additionally, participants may have made some progress towards achieving their goals, but the dichotomous recording of goal achievement in this study meant that partial goal achievement would have been recorded as ‘No’, which was a further limitation. Further work is needed to identify how to optimise goal setting from the perspectives of patients and therapists, especially for those with severe arm impairment and an unfavourable prognosis, to ensure that goals are meaningful and achievable.

A further factor related to goal achievement is the therapy dose, on which the literature is currently unclear.^43^ Dose is a multi-factorial concept which includes the frequency, intensity, duration and timing of an intervention. Often, studies of dose in rehabilitation trials focus on therapy time only, however, in the RATULS trial we have been able to report the actual use of therapy time. The optimum amount of additional therapy time needed to improve arm activity limitation after stroke is the subject of debate. A 2014 meta-analysis found strong evidence that an additional 17 hours of physiotherapy significantly improved a range of outcomes, including arm function, basic ADL and quality of life after stroke.^17^ A 2014 Cochrane overview and a 2016 Cochrane systematic review reported that repetitive task training improved upper limb function, but there were no significant differences in outcomes between a dose of at least 20 hours compared with smaller doses.^3,5^ A number of more recent randomized controlled trials of upper limb therapy post-stroke have also been neutral for their primary outcome.^43–48^ These trials planned to deliver between 10 and 45 hours of additional upper limb therapy, with four trials aiming to deliver an additional 30 hours or more.^43,44,46,48^ It has been suggested that the amount of therapy time provided to both intervention groups (robot-assisted training and enhanced upper limb therapy) in the RATULS trial was too low,^49,50^ even though benefit was seen for a number of secondary outcomes at the end of the three month intervention period when the enhanced upper limb therapy programme was compared with usual care. Three studies have reported benefits of high amounts of therapy time (i.e. 300 hours^51,52^ and 90 hours^53^) in upper limb therapy for chronic stroke patients – with less severe initial arm activity limitation – however, none of these studies had a lower dose or usual care comparator. Whilst these results are interesting, these interventions require further evaluation in robust, adequately powered randomized controlled trials to demonstrate their clinical effectiveness and cost-effectiveness.

Results from studies which aimed to determine the optimum dose in terms of repetitions have been inconsistent and inconclusive so far. A Phase II single-blind, randomized, repetition dose response study evaluated a task-specific upper limb rehabilitation intervention comprising up to 32 hours of practice for stroke survivors at least six months after stroke with mild to moderate arm impairment.^54^ Eighty-five participants were randomized to undertake either 3200, 6400, 9600, or individualized maximum repetitions. All groups improved but there was no evidence of a dose-response relationship between the number of repetitions and outcome. Our findings also suggest that the relationship between the number of repetitions undertaken and the proportion of goals achieved is unclear, as the number of repetitions completed by those with the lowest proportion of goals achieved (i.e. those who were more than 12 months post stroke with Action Research Arm Test scores 0–7) is not dissimilar to that completed by those with the highest proportion of goals achieved (i.e. anyone less than 3 months post stroke irrespective of their Action Research Arm Test scores). Further research is needed to analyse this relationship. The lack of relationship between number of repetitions and outcome raises the question about what is being repeated in a repetition, and whether this actually facilitates skill acquisition. In addition to duration and number of repetitions, the content and scheduling of therapy also need to be considered. A 2010 systematic review of task-orientated training to improve arm and hand performance after stroke identified that the following skill acquisition components were associated with better outcomes: clear functional goals, variety, distributed practice (where the amount of rest exceeds the amount of practice), random practice (where activities are practised in random order), context-specific practice (where the practice environment simulates real-life) and feedback.^55^ Our enhanced upper limb therapy programme included clear functional goals with task variety and random practice, however the distribution between practice and rest, and the type and timing of feedback were not protocolised nor recorded. Whilst context-specific practice was attempted, the hospital environment inevitably imposed some limitations. These aspects could be considered for future studies.

This study has a number of strengths and limitations, in addition to those already discussed. Generalization of the findings from this study to the general stroke population with moderate to severe arm activity limitation is limited by the finding that RATULS trial participants were younger than the average stroke population in the UK (60 years vs 75 years) and comprised more males (61% vs 50%).^56^ The fact that individuals with moderate to severe arm activity limitation were recruited up to five years after stroke meant that a considerable proportion of participants had a poor prognosis for recovery.^36^ Furthermore, outcomes may have been influenced by self-practice, undertaken by participants outside of formal trial intervention sessions. Although self-report forms indicated a similar high level of self-practice for all randomization groups across the study period,^31^ information about content and dose of self-practice was not collected. Therefore it is not possible to comment on the impact of this potential confounding factor. The strengths of this study are that training, documented in study manuals, was provided in delivery and recording of the enhanced upper limb therapy programme, which could be replicated in clinical practice or further developed in future studies.

**Clinical messages**

- The enhanced arm therapy programme for stroke patients with moderate-to-severe arm activity limitation can be delivered by supervised physiotherapy assistants with high fidelity.
- The most common goals focused on eating/drinking, range of movement, and dressing.
- The proportion of goals achieved varied according to time post stroke and baseline arm activity level.

## Supplemental Material

EULT_manuals_070220 – Supplemental material for Evaluation of the enhanced upper limb therapy programme within the Robot-Assisted Training for the Upper Limb after Stroke trial: descriptive analysis of intervention fidelity, goal selection and goal achievement Click here for additional data file. Supplemental material, EULT_manuals_070220 for Evaluation of the enhanced upper limb therapy programme within the Robot-Assisted Training for the Upper Limb after Stroke trial: descriptive analysis of intervention fidelity, goal selection and goal achievement by Helen Bosomworth, Helen Rodgers, Lisa Shaw, Leanne Smith, Lydia Aird, Denise Howel, Nina Wilson, Natasha Alvarado, Sreeman Andole, David L Cohen, Jesse Dawson, Cristina Fernandez-Garcia, Tracy Finch, Gary A Ford, Richard Francis, Steven Hogg, Niall Hughes, Christopher I Price, Laura Ternent, Duncan L Turner, Luke Vale, Scott Wilkes, Hermano I Krebs and Frederike van Wijck in Clinical Rehabilitation
