# Supplementary material for: Evaluation of the enhanced upper limb therapy programme within the Robot-Assisted Training for the Upper Limb after Stroke trial: descriptive analysis of intervention fidelity, goal selection and goal achievement
Source: Clin Rehabil. 2020 Sep 11;35(1):119–34. doi: 10.1177/0269215520953833 (PMC7814096; doi:10.1177/0269215520953833)
Supplement: EULT_manuals_070220 – Supplemental material for Evaluation of the enhanced upper limb therapy programme within the Robot-Assisted Training for the Upper Limb after Stroke trial: descriptive analysis of intervention fidelity, goal selection and goal achievement [file EULT_manuals_070220.pdf]

National Institute for Health Research

Newcastle University

Robot Assisted Training for the Upper Limb after Stroke

RATULS

Enhanced upper limb therapy 1: **Programme overview**

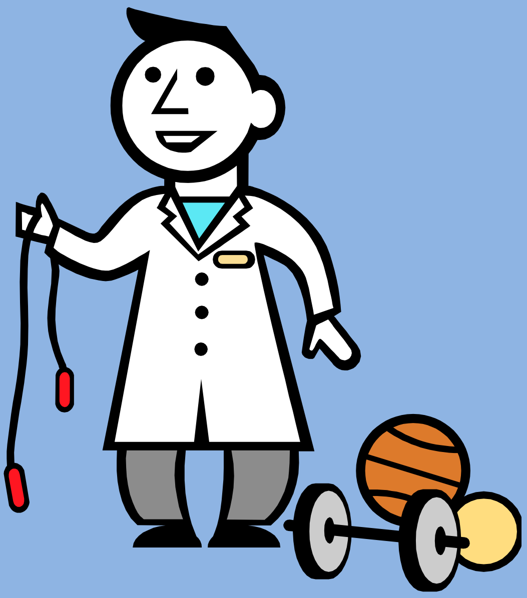

© RATULS study team 2015

Contents

1. Purpose of this document 3

2. Introduction 3

3. Summary of the enhanced upper limb therapy programme 4

4. Enhanced upper limb therapy staff roles and responsibilities 6

5. RATULS enhanced therapy summary diagram 7

6. Other important points about the RATULS enhanced therapy programme 8

7. Enhanced upper limb therapy documents 9

8. RATULS co-ordinating centre 9

9. References 9

© RATULS study team 2015

1. Purpose of this document

This document gives an overview of the enhanced upper limb therapy programme being used in the RATULS study. It is intended to serve as a reference and resource guide for clinicians delivering the enhanced upper limb therapy programme.

2. Introduction

The RATULS study is a three group multicentre randomised controlled trial to determine whether robot assisted training improves upper limb function after stroke. Robot assisted training is being compared to i) an enhanced upper limb therapy programme consisting of repeated practise of everyday activities using the arm and ii) usual NHS rehabilitation. Stroke patients with reduced arm function who wish to take part in the trial are randomly assigned to either robot assisted training, enhanced upper limb therapy or usual NHS rehabilitation.

The RATULS enhanced upper limb therapy programme consists of task-orientated practice aimed at participant-centred goals. It has been designed to facilitate upper limb function, reduce learned non-use of the affected arm and reduce arm impairments that are commonly found in people who have had a stroke.

The programme has been developed from upper limb therapy programmes used in the Botulinum Toxin for the Upper Limb after Stroke (BoTULS) trial[1, 2] and the Repetitive Arm Functional Tasks after Stroke (RAFTAS) project[3].

© RATULS study team 2015

3. Summary of the enhanced upper limb therapy programme

The RATULS enhanced upper limb therapy programme consists of three 45 minute therapy sessions per week for 12 weeks. One hour has been allocated for each session to allow for set-up, preparation and completion of study documents. The total number of therapy sessions per participant is 36. In each therapy session, participants practise activities to work towards their upper limb rehabilitation goals. Upper limb rehabilitation goals are selected at the initial therapy session and reviewed/adjusted at sessions 12 (end of week 4) and 24 (end of week 8).

Types of goals/activities

To facilitate delivery of the RATULS enhanced upper limb therapy programme, a list of potential goals and a description of suggested activities for each goal, has been prepared. Whilst it is anticipated that this will cover many participant choices, it is acceptable for alternative goals and activities to be determined and used the discretion of the local therapist. The prepared goals/activities cover washing; dressing; eating and drinking and 'other' categories.

Types of practice

Activities are divided into two types: 'whole-task' or a 'part-task'. Whole-task activity practice consists of practising all of the components of the task in sequence. Part-task activity practice consists of practicing a specific part of a task. Part-task practice is appropriate if a participant has difficulty with a specific part of a task as it will enable them to focus on this particular aspect independently to completing the task as a whole. The 'whole-task' and 'part-task' activities prepared for RATULS are described in a series of flowcharts which show how to complete each activity step by step. Choice of a whole-task or part-task activity for each goal will be dependent on participant ability. Following accomplishment of a 'part-task' activity participants progress to practise the next 'part-task' activity in the step by step flowchart. Should a 'whole-task' activity be accomplished before a goal review session, the flowcharts include suggestions for adaptations to the activities for further practice.

Intensity of practice

To achieve functional improvement after stroke, a few hundred repetitions are required each session[4]. Therefore, in this study we should aim – where possible – to include a few hundred repetitions in each session.

© RATULS study team 2015

What is a repetition?

- For whole task practice, completion of the whole task (from the beginning to the end of the flowchart) counts as one repetition, i.e. from the start position to a return to the start position or to completion of the task (if different from the start position).
- For part task practice completion of the *component* of the task counts as one repetition.

(Note: a continuous task (e.g. walking while carrying a plate) should be interpreted as a discrete task for the upper limb and repetitions should be counted as such (e.g. picking a plate up, carrying it and putting it down again = 1 repetition).

<u>Working through each session</u>

At the initial therapy session, up to four upper limb rehabilitation goals of importance to the participant will be agreed and the activities to practise to achieve these goals subsequently determined. Participants will then undertake a brief warm up consisting of gentle stretching of the upper limb, prior to practice of the chosen activities. The order to practise the activities and the time to spend on each activity will be at the discretion of the therapist and participant according to the participant's rehabilitation priorities.

At the second and subsequent therapy sessions, following a brief warm up (if necessary), practice of the selected activities will continue, with the order to practise and time to spend on each activity being at local discretion. At therapy sessions 12 (end of week 4) and 24 (end of week 8), progress towards goals will be reviewed. If the participant has achieved a goal, a new goal will be set and a new activity to practise selected. If the participant is finding a goal or activity too challenging or they are experiencing other problems, an alternative will be chosen.

At the final therapy session (36, end of week 12), practice of activities will continue but part of the session will also be dedicated to 'summing up' with feedback to the participant about progress over the programme and advice about maintaining upper limb function in the longer term. There will also be an opportunity for participants to give views on the therapy programme.

© RATULS study team 2015

4. Enhanced upper limb therapy staff roles and responsibilities

The enhanced upper limb therapy programme is intended to be delivered by therapists appointed or seconded to work with a RATULS study centre team.

It is intended that day to day therapy sessions are delivered by a therapy assistant with supervision and advice from a senior therapist. The senior therapist is also responsible for regular participant reviews.

Senior Therapist:

The senior therapist is responsible for:

- Week 1 (session 1):
  - Initial assessment, goal setting and activity choice
  - Supervision of the warm up stretching (if necessary)
  - Supervision of initial activity practice.
- Week 4 and 8 (sessions 12 and 24):
  - Evaluation of progress
  - Upper limb reassessment and review of goals.
  - Setting new goals (where appropriate)
  - Choosing new activities to practise (where appropriate).
- Week 12 (session 36):
  - Giving feedback about progress
  - Giving advice about maintaining upper limb function in the longer term.
- Supervision and support for the therapy assistant throughout the programme
- Completing relevant sections of the enhanced therapy programme paperwork
- Ensuring that the enhanced upper limb therapy programme is delivered as per protocol.

Therapy Assistant:

The therapy assistant is responsible for:

- Day-to-day enhanced upper limb therapy sessions
- Reporting any concerns about participants to the senior therapist
- Acting on advice given by the senior therapist
- Completing relevant sections of the enhanced therapy programme paperwork
- Ensuring that the enhanced upper limb therapy programme is delivered as per protocol.

© RATULS study team 2015

5. RATULS enhanced therapy summary diagram

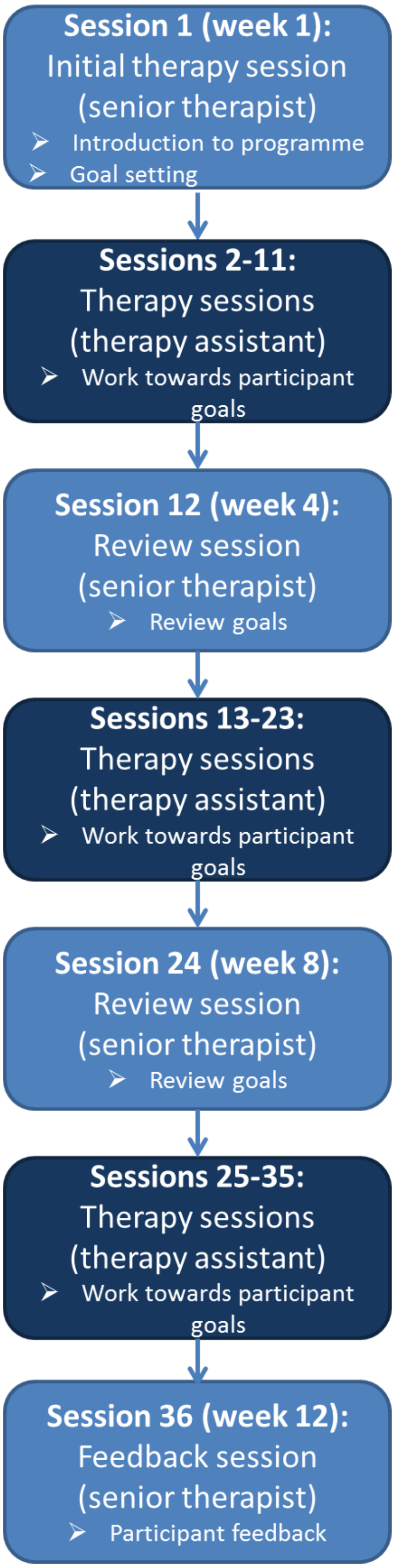

© RATULS study team 2015

6. Other important points about the RATULS enhanced therapy programme

The RATULS enhanced upper limb therapy programme is based on the following principles:

It should be:

- Relevant for participants
- Challenging
- Achievable
- Promote long-term benefits (as opposed to short term performance benefits)
- Engaging
- Standardised in terms of treatment principles, while the content should allow some tailoring to individual participants' goals and abilities.

It is acknowledged that the programme is somewhat prescriptive and that a personal, tailor-made programme would be more representative of normal clinical practice. However, within the context of this research project, the benefits and drawbacks of an individual approach had to be balanced against the need to avoid potential confounding variables and also to record the therapeutic input in sufficient detail to enable replication of the study. Therefore, principles of the programme have been standardised, whilst leaving sufficient opportunity for specific task requirements to be fine-tuned to the capabilities and goals of each individual participant.

© RATULS study team 2015

7. Enhanced upper limb therapy documents

This Enhanced upper limb therapy 1: Programme overview manual is part of a series of documents developed to describe the RATULS enhanced upper limb therapy programme. The other documents in this series are:

- Enhanced upper limb therapy 2: How to deliver the programme
- Enhanced upper limb therapy 3: Warm-up stretches, goal choices and activity flowcharts

8. RATULS co-ordinating centre

For further information please contact the RATULS co-ordinating team:

Stroke Research Group

3-4 Claremont Terrace

Newcastle University

Newcastle Upon Tyne

NE2 4AE

Email: ratuls@ncl.ac.uk

Phone: 0191 2086779

Or refer to the RATULS website: http://research.ncl.ac.uk/ratuls/

9. References

1. Shaw, L., H. Rodgers, C. Price, F. van Wijck, P. Shackley, N. Steen, M. Barnes, G. Ford, and L. Graham, Health Technol Assess, BoTULS: a multicentre randomised controlled trial to evaluate the clinical effectiveness and cost-effectiveness of treating upper limb spasticity due to stroke with botulinum toxin type A. 2010. **14**(26): p. 1-113, iii-iv.
2. Shaw, L.C., C.I. Price, F.M. van Wijck, P. Shackley, N. Steen, M.P. Barnes, G.A. Ford, L.A. Graham, and H. Rodgers, Stroke, Botulinum Toxin for the Upper Limb after Stroke (BoTULS) Trial: effect on impairment, activity limitation, and pain. 2011. **42**(5): p. 1371-9.
3. Brkic L., Shaw L., F. van Wijck, Price C., C. Watkins, A. Forster, P. Langhorne, and H. Rodgers, *Repetitive arm functional tasks after stroke (RAFTAS) study Therapy Manual*, in *The Stroke Association Project Ref No: 2010/05*2013.
4. Birkenmeier, R.L., E.M. Prager, and C.E. Lang, Neurorehabil Neural Repair, Translating animal doses of task-specific training to people with chronic stroke in 1-hour therapy sessions: a proof-of-concept study. 2010. **24**(7): p. 620-35.

© RATULS study team 2015

This enhanced upper limb therapy manual has been produced by Dr Helen Bosomworth with input from Miss Lianne Brkic, Mrs Lydia Aird, Dr Lisa Shaw and Professor Frederike van Wijck.

© RATULS study team 2015:
Helen Rodgers, Newcastle University
Lydia Aird, Northumbria Healthcare NHS Foundation Trust
Sreeman Andole, Barking, Havering & Redbridge University Hospitals NHS Trust
David Cohen, London North West Hospitals NHS Trust
Jesse Dawson, University of Glasgow
Janet Eyre, Newcastle University
Tracy Finch, Newcastle University
Gary Ford, Newcastle University
Steven Hogg, lay member
Denise Howel, Newcastle University
Niall Hughes, NHS Greater Glasgow and Clyde
Hermano Igo Krebs, Massachusetts Institute of Technology
Chris Price, Northumbria Healthcare NHS Foundation Trust
Lynn Rochester, Newcastle University
Lisa Shaw, Newcastle University
Laura Ternent, Newcastle University
Duncan Turner, University of East London
Luke Vale, Newcastle University
Frederike van Wijck, Glasgow Caledonian University
Scott Wilkes, University of Sunderland

© RATULS study team 2015

Robot **A**ssisted **T**raining for the **U**pper **L**imb after **S**troke

RATULS

Enhanced upper limb therapy 2: **How to deliver the programme**

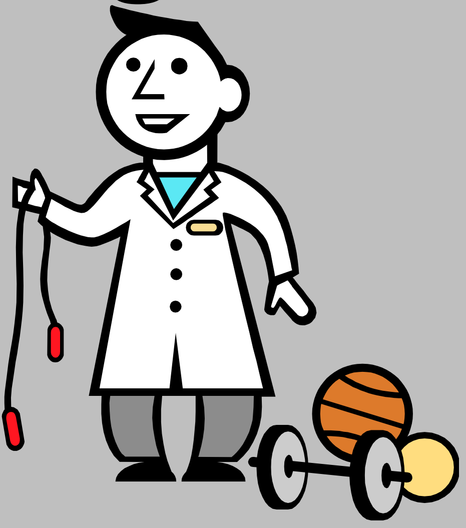

© RATULS study team 2015

Contents

1. Purpose of this document 4

2. Initial therapy session (session 1) 4

2.1. Introduction 4

2.2. Assessment 4

2.3. Warm-up stretching 5

2.4. Core treatment 6

2.5. Summary and feedback 8

2.6. Study paperwork 8

3. Four week and eight week review sessions (sessions 12 and 24) 11

3.1. Introduction 11

3.2. Reassessment 11

3.3. Warm-up stretching 12

3.4. Core treatment 12

3.5. Summary and feedback 12

3.6. Study paperwork 13

4. Final therapy session (session 36) 14

4.1. Introduction 14

4.2. Warm-up stretching 14

4.3. Core treatment 14

4.4. Summary and feedback 14

4.5. Study paperwork 15

© RATULS study team 2015

5. Therapy sessions 16
5.1. Introduction 16
5.2. Warm-up stretching 16
5.3. Core treatment 17
5.4. Summary and feedback 21
5.5. Study paperwork 21

6. Senior therapist session summaries 22
6.1. Session 1: initial therapy session 22
6.2. Sessions 12 and 24: Review sessions 23
6.3. Session 36: Final therapy session 24

7. Therapy assistant session summaries 25

8. Recording therapy - participant goal and therapy record 26

9. Participant attendance 38

10. Enhanced upper limb therapy documents 40

11. RATULS co-ordinating centre 40

12. References 40

© RATULS study team 2015

1. Purpose of this document

This document describes how to deliver the enhanced upper limb therapy programme being used in the RATULS study. It is intended to serve as a reference and resource guide for clinicians delivering the enhanced upper limb therapy programme. For a summary of the RATULS enhanced upper limb therapy programme, see ‘Enhanced upper limb therapy 1: programme overview’.

2. Initial therapy session (session 1)

The first session of the enhanced upper limb therapy programme covers an initial assessment followed by goal setting/activity choice and initial therapy. It should be carried out by a senior therapist in conjunction with a therapy assistant.

2.1. Introduction

The therapy session should begin with a description of what will be involved in the RATULS enhanced upper limb therapy programme.

2.2. Assessment

A standard upper limb assessment should be completed to establish motor impairment and other neurological deficits that may impact on the participant’s upper limb function. The therapist should pay particular attention to:

- Selective movement
- Passive range of movement
- Muscle tone
- Proprioception
- Sensation
- Compensations
- Pain
- Coordination
- Associated reactions

In addition, any inattention should be noted.
A discussion of the participant’s upper limb rehabilitation needs and identification of important goals should follow. Up to four rehabilitation goals should be agreed.

© RATULS study team 2015

Suggested goal choices can be found in ‘Enhanced upper limb therapy 3: warm up stretches, goal choices and activity flowcharts’. Whilst it is anticipated that this goal list will cover many participant choices, it is acceptable for alternative goals to be set at local discretion by the senior therapist.

Following agreement of up to four goals, activities to practise should be determined. For goals chosen from the enhanced therapy list, associated ‘whole-task’ and ‘part-task’ activity choices are provided in step by step flowcharts. Whole-task activity practice consists of practising all the components of the task in sequence. Part-task activity practice consists of practising a specific part of a task. Part-task practice is appropriate if a participant has difficulty with a specific part of a task as it will enable them to focus on this particular aspect independently to completing the task as a whole. Choice of a whole-task or part-task activity for each goal is therefore dependent on participant ability. If any activities within the whole-task or part-task flowcharts are not suitable for a participant, they can be adapted at the discretion of a senior therapist.

If a goal is chosen which is not included within the enhanced therapy goal list, it will be necessary for a senior therapist to give appropriate advice on activities to practise for this goal, to the therapy assistant.

2.3. Warm-up stretching

Gentle stretching of soft tissues and mobilisation of joints can be performed, if necessary, to focus attention on the affected upper limb prior to practising rehabilitation activities. However, as the focus of the therapy sessions should be activity practice working towards the participant goals, warm-up stretching should be kept to a minimum. Example warm up stretches can be found in ‘Enhanced upper limb therapy 3: Warm-up stretches, goal choices and activity flowcharts’. Different stretches may be used at the discretion of a senior therapist. Warm up stretches should be demonstrated to both the participant and the therapy assistant.

© RATULS study team 2015

2.4. Core treatment

A short therapy session to introduce both the participant and the therapy assistant to the tasks the participants will be working through in the following 4 weeks, should next be undertaken.

The senior therapist should ensure that both the therapy assistant and participant are familiar with the tasks by demonstration and supervision of practice. In this session and other subsequent therapy sessions, the order to practise the selected tasks and the time to spend on each task are at local discretion and according to a participant’s rehabilitation priorities.

During activity practice, the number of repetitions of each task need to be counted and recorded.

What is a repetition?

- For whole task practice, completion of the whole task (from the beginning to the end of the flowchart) counts as one repetition, i.e. from the start position to a return to the start position or to completion of the task (if different from the start position).
- For part task practice completion of the *component* of the task counts as one repetition.

(Note: a continuous task (e.g. walking while carrying a plate) should be interpreted as a discrete task for the upper limb and repetitions should be counted as such (e.g. picking a plate up, carrying it and putting it down again = 1 repetition).

Number of repetitions should be recorded within the ‘Enhanced upper limb therapy: participant goal and therapy record’.

Can stretching count as activity practice?

If a ‘stretch’ is embedded into an active task (e.g. reaching out to touch/ grasp an object), the repetitions of that stretch/task should be recorded as activity practice. However, if a stretch is passive (i.e. this is not a voluntary, active action on behalf of the patient but a manoeuvre undertaken by the therapist) this does not count as activity practice and the repetitions should NOT be recorded.

© RATULS study team 2015

In the RATULS enhanced upper limb therapy programme, the senior therapist goal review sessions are at the end of week 4 (session 12) and end of week 8 (session 24). This means that appropriate guidance regarding progression of therapy will need to be provided for a therapy assistant delivering the therapy sessions three times per week.

Where activities to practise have been chosen from the 'Enhanced upper limb therapy 3: warm up stretches, goal choices and activity flowcharts', the activities are designed such that following accomplishment of a 'part-task' activity participants progress to practise the next 'part-task' activity in the step by step flowchart. Should a 'whole-task' activity be accomplished before a goal review session, the flowcharts include suggestions for adaptations to the activities for further practise and progression. If a goal is chosen which is not included within the enhanced therapy goal list, it will be necessary for the senior therapist to give appropriate advice on activities to practise and how to progress therapy, to the therapy assistant.

In addition, the following should be considered and appropriate guidance given regarding practising activities over the next four weeks:

For each activity, once the participant knows what to do and they demonstrate a basic ability, try to make the activity more engaging and stimulating, e.g. by:

- Including *variations* in the task (e.g. different sizes and shapes of objects, different start-and end positions, different speeds). This is known as variable practice. Variable practice enables the participant to translate what they learn during therapy sessions more easily to a wider range of activities of daily living (ADL).
- Doing tasks in a *different order* within a therapy session. So rather than doing five repetitions of one activity followed by five repetitions of another task and so on (known as blocked practice), mix up the order in which the participant undertakes each task (known as random practice). This may seem counter-intuitive, but random practice encourages deeper learning. Random practice also allows for a more 'real-life' situation where the order of events is more

© RATULS study team 2015

likely to be mixed rather than blocked. This enables better translation of what a participant has learned during therapy into ADL.

Ensure the programme is sufficiently intensive. To achieve functional improvement after stroke, a few hundred repetitions are required each session[1]. Therefore, in this study we should aim – where possible – to include a few hundred repetitions in each session.

2.5. Summary and feedback

This should consist of a participant's own evaluation of their performance followed by therapist feedback. This discussion is to encourage a participant to think about what they have learned, and what they need to focus on in the next session.

2.6. Study paperwork

Accurate completion of paperwork is a crucial part of research.

All sessions should be recorded in the document titled 'Enhanced upper limb therapy: participant goals and therapy record'. In addition, a second document entitled 'Enhanced upper limb therapy: activity flowchart for optional goals or activity adaptations' exists to record activities for optional goals and any adaptions to the pre-specified activity flowcharts (found in 'Enhanced upper limb therapy 3: warm up stretches, goal choices and activity flowcharts'). Section 6 below shows how these documents should be completed. In summary, the following must be documented for session 1:

- the (up to four) goals that have been selected
    - if a goal has been selected from the pre-specified list, please document this by its number as listed in 'Enhanced upper limb therapy 3: warm up stretching, goal choices and activity flowcharts' (e.g. D1).
    - If an alternative goal has been chosen ('optional goal'), this should be written out free text and labelled as 'OG 1' (optional goal 1). If more than one optional goal has been chosen these should also be written

© RATULS study team 2015

out and then labelled as OG 2, OG 3, etc. For example: ‘Using a knife and fork – OG1’

- whether whole-task of part-task activity practice has been chosen, for each goal
- where part-task practice has been chosen for a pre-specified goal, the start position on the relevant flowchart should be recorded (indicated by the number in the flowchart box e.g. W1.02 (see ‘Enhanced upper limb therapy 3: warm up stretching, goal choices and activity flowcharts’)
- where an optional goal has been chosen details of the activities to practice, the starting task and tasks for progression should be recorded (this should be in the document entitled ‘Enhanced upper limb therapy: activity flowchart for optional goal or goal adaptations’)
- any adaptations to the pre-specified activity flowcharts which are being made (this should be in the document entitled ‘Enhanced upper limb therapy: activity flowchart for optional goal or goal adaptations’)
- any guidance about progression for the pre-specified activity flowcharts should be detailed in the session notes. Where an optional goal is being used or pre-specified activities have been adapted, progression guidance should be documented on the document entitled ‘Enhanced upper limb therapy: activity flowchart for optional goals or activity adaptations’.
- the therapy record which includes
  - the goals worked on (record as D1, OG1, etc)
  - the number of repetitions of the task which has been practised for each goal. The total number of repetitions should be recorded.
  - the duration of the entire session
  - the duration of activity practice
  - the treating senior therapist’s name, and signature
  - the date
  - a notes section to record any important aspects of therapy, e.g. if a participant has achieved a part-task activity and should commence at the next part-task in the flowchart in the next therapy session, this should be recorded here.

© RATULS study team 2015

The ‘Enhanced upper limb therapy: participant goal and therapy record’ also includes an optional section to record an upper limb assessment, should this be useful to the senior therapist.

© RATULS study team 2015

3. Four week and eight week review sessions (sessions 12 and 24)

Review sessions should be carried out at the end of week 4 (session 12) and the end of week 8 (session 24). The aim of the review sessions is to assess a participant's progress with the programme, and review goals and activities. The review sessions should be carried out by a senior therapist in conjunction with a therapy assistant.

3.1. Introduction

The session should begin with a discussion about progress towards the goals and practising the activities to date.

3.2. Reassessment

An upper limb reassessment should be completed to establish current motor impairment and other neurological deficits that may impact on the participant's upper limb function. The therapist should pay particular attention to:

- Selective movement
- Passive range of movement
- Muscle tone
- Proprioception
- Sensation
- Compensations
- Pain
- Coordination
- Associated reactions

In addition, any inattention should be noted.

A review of upper limb rehabilitation goals should take place. New goals may be selected if previous goals have been achieved, or existing goals may be modified e.g. increasing/decreasing difficulty. Activities choices should also be reviewed accordingly e.g. this may include change from part task practice to whole task practice.

© RATULS study team 2015

3.3. Warm-up stretching

Gentle stretching of soft tissues and mobilisation of joints can be performed, if necessary, prior to practising rehabilitation activities. However, as the focus of the therapy sessions should be activity practice working towards the participant goals, warm-up stretching should be kept to a minimum. Stretches may be modified at the discretion of a senior therapist. Warm up stretches should be demonstrated to both the participant and the therapy assistant.

3.4. Core treatment

A short therapy session to introduce both the participant and the therapy assistant to the revised tasks the participants will be working through in the following four weeks, should next be undertaken.

The senior therapist should ensure that both the therapy assistant and participant are familiar with the tasks by demonstration and supervision of practice. Guidance on how to progress tasks over the next four weeks should also be given.

As previously, the order to practise the selected tasks and the time to spend on each task are at local discretion and according to a participant's rehabilitation priorities. Repetitions of tasks need to be counted and recorded.

3.5. Summary and feedback

This should consist of a participant's own evaluation of their performance followed by therapist feedback. This discussion is to encourage a participant to think about what they have learned, and what they need to focus on in the next session.

© RATULS study team 2015

3.6. Study paperwork

The ‘Enhanced upper limb therapy: participant goal and therapy record’ and ‘Enhanced upper limb therapy: activity flowchart for optional goals or activity adaptions’ should be completed as appropriate.

The following must be documented:

- whether a participant has achieved their goals
- the (up to four) goals to work towards
- whole-task/part task activity choice (including completion of additional activity flowcharts using ‘Enhanced upper limb therapy: activity flowchart for optional goals or activity adaptations’ as appropriate)
- any further information/guidance (included in the session notes or on the activity flowchart for optional goals or activity adaptations).
- the therapy record which includes:
    - the goals worked on
    - the number of repetitions of the task which has been practised for each goal
    - the duration of the entire session
    - the duration of activity practice
    - the treating senior therapist’s name, and signature
    - the date
    - any notes.

© RATULS study team 2015

4. Final therapy session (session 36)

The final therapy session should be performed by a senior therapist in conjunction with a therapy assistant. The aim of this session is to conclude the RATULS enhanced upper limb therapy programme. It includes further activity practice followed by feedback and advice for the future.

4.1. Introduction

The session should begin with a discussion about progress towards the goals and practising the activities to date.

4.2. Warm-up stretching

As previously, warm up stretches can be performed where necessary before practising activities.

4.3. Core treatment

Final activity practice should take place focussing on any areas of particular importance to the participant. Suggestions for activities to practise at home or advice about further rehabilitation services can be made if appropriate. Advice about maintaining current upper limb function in the longer term should also be included.

4.4. Summary and feedback

A final feedback discussion should be held. This should include discussion of a participant's own evaluation of their performance and therapist feedback.

Feedback on the thoughts on the content and structure of the enhanced upper limb therapy programme is sought from the participant as part of the enhanced upper limb rehabilitation therapy log.

© RATULS study team 2015

4.5. Study paperwork

The appropriate sections in the ‘Enhanced upper limb therapy: participant goal and therapy record’ must be completed.

The following must be documented:

- whether a participant has achieved their goals
- the therapy record which includes:
  - the goals worked on
  - the number of repetitions of the task which has been practised for each goal
  - the duration of the entire session
  - the duration of activity practice
  - the treating senior therapist’s name, and signature
  - the date
  - any notes.
- feedback given to participant about progress over the programme
- further advice given regarding maintaining upper limb function (e.g. home exercises).

© RATULS study team 2015

5. Therapy sessions

Excluding the sessions discussed above, there are 32 other therapy sessions within the enhanced upper limb therapy programme. These sessions are designed to be carried out by a therapy assistant but can be conducted by a qualified therapist according to local staff availability. The aim of these therapy sessions is to practise activities to work towards the goals selected at the initial therapy session or the therapy review sessions.

5.1. Introduction

At the beginning of each therapy session, the therapy assistant should review a participant's previous session notes as a reminder of the goals and activities selected and progress to date.

A discussion should be held regarding which of the selected goals/activities the participant would like to work on at this session. The order to practise each activity and the time to spend on each activity is according to local discretion, A participant need not practise the activities for all four chosen goals in each session.

5.2. Warm-up stretching

Gentle stretching of soft tissues and mobilisation of joints can be performed, if agreed at the initial/ review sessions, to focus attention on the affected upper limb prior to practising rehabilitation activities. However, as the focus of the therapy sessions should be activity practice working towards the participant goals, warm-up stretching should be kept to a minimum. Example warm up stretches can be found in 'Enhanced upper limb therapy 3: Warm-up stretches, goal choices and activity flowcharts'.

© RATULS study team 2015

5.3. Core treatment

Following warm up stretching (where appropriate), practise of selected activities should commence. The activity flowcharts in 'Enhanced upper limb therapy 3: Warm-up stretches, goal choices and activity flowcharts' or the 'Enhanced upper limb therapy: activity flowchart for optional goals or activity adaptations' should be used to guide practice. The flowcharts are designed as a therapy aid rather than a definitive instruction on how to provide therapy. Guidance and advice from the senior therapist should always be followed.

The following principles should also be considered when delivering therapy:

1. **Demonstration**

Explain a task to the participant and show them first what to do. Then ask them to undertake/ lead the task with their non-affected side. This is to ensure that the participant has a clear understanding of what to do before they start repeating it with their more affected side. This also enables the non-affected arm to "teach" the affected arm. Following this practise with the non-affected side, commence practice with the affected side.

2. **Progressing practice**

The RATULS enhanced upper limb therapy programme is split into three, 4 week therapy blocks. To enable participants to achieve the goals set at the beginning of a block, it is important to progress treatment; mindless repetition is not effective.

For part-task activities, once the part task has been accomplished, the participant should progress onto the next part-task in the flowchart.

For whole task activities, the flowcharts also include suggestions for modifications to the activities for further practice and progression.

In addition, the following should be considered:
For each activity, once the participant knows what to do and they demonstrate a basic ability, try to make the activity more engaging and stimulating, e.g. by:

© RATULS study team 2015

- Including *variations* in the task (e.g. different sizes and shapes of objects, different start-and end positions, different speeds). This is known as variable practice. Variable practice enables the participant to translate what they learn during therapy sessions more easily to a wider range of ADL.
- Doing tasks in a *different order* within a therapy session. So rather than doing five repetitions of one activity followed by five repetitions of another task and so on (known as blocked practice), mix up the order in which the participant undertakes each task (known as random practice). This may seem counter-intuitive, but random practice encourages deeper learning. Random practise also allows for a more 'real-life' situation where the order of events is more likely to be mixed rather than blocked. This enables better translation of what a participant has learned during therapy into ADL.

3. Intensity of practice

Ensure the programme is sufficiently intensive. To achieve functional improvement after stroke, a few hundred repetitions are required each session[1]. Therefore, in this study we should aim – where possible – to include a few hundred repetitions in each session.

4. Monitoring compensatory movements

It is important to monitor so-called "compensatory movements" during practice. These are movements that compensate for those that the participant finds difficult. The reasons why some movements may be difficult include muscle weakness, stiffness, pain or a mix of these. Compensatory movements are often unintentional and people are often unaware that they are "compensating". Compensatory movements tend to be used in order to achieve a goal (e.g. reach for an object). Common compensatory movements when using the affected arm after stroke are:

- Moving the trunk forward or sideways when reaching. This may be due to difficulty lifting the arm forwards or sideways, and/or difficulty extending the elbow and/or wrist.
- Lifting the shoulder girdle on the affected side when reaching with the affected arm. The participant may attempt to complete a task by lifting the shoulder girdle instead of their arm.

© RATULS study team 2015

Why is it important to monitor compensatory movements? When practising a task, it is not only about how many repetitions, but also about how well they are executed. In order to achieve the best outcomes, participants need to practise those movement(s) that they find difficult and minimise compensatory movements. Using compensatory movements may mean that a participant does not optimally practice the movement(s) they find difficult.

How to avoid compensatory movements? Before a participant starts to practise a task, it is important to remind them of the optimal posture for practice. Instructions such as "keep your body upright" (to avoid compensatory trunk movement), "keep your shoulders down" (to avoid compensatory shoulder girdle movement) may help. Should compensatory movements arise during practice, a participant should be made aware of these before they attempt again. In some cases, a task may need to be adapted (e.g. a shorter reach, a lighter object) if compensatory movements continue. However, some compensatory movements are likely to take place as practice is meant to be difficult, and with training they are likely to diminish.

5. Equipment

Many activities involve the use of functional objects (e.g. mug, comb, item of clothing). This enhances carry-over of the practice into real life. Where chosen activities require items individual to a participant, they should be asked to bring in their items to use (e.g. comb/brush, specific garments, adapted cutlery). More generic objects should be available in the routine therapy setting (including mugs, cups, containers etc.).

Recording activity practice

During activity practice, the number of repetitions of each task needs to be counted and recorded:

- For whole task practice, completion of the whole task (beginning to end of flowchart) counts as one repetition.
- For part task practice completion of the *component* of the task counts as one repetition.

© RATULS study team 2015

- If a mixture of whole and part task practice is being used then count the repetitions as above and add the repetitions for each task together to give a total for that goal.

Can stretching count as activity practice?

If a ‘stretch’ is embedded into an active task (e.g. reaching out to touch/ grasp an object), the repetitions of that stretch/task should be recorded as activity practice. However, if a stretch is passive (i.e. this is not a voluntary, active action on behalf of the patient but a manoeuvre undertaken by the therapist) this does not count as activity practice and the repetitions should NOT be recorded.

© RATULS study team 2015

5.4. Summary and feedback

At the end of each session, a participant should be asked for comments on their performance and the therapist should also give some feedback. This discussion is to encourage the participant to think about what they have learned, and what they need to focus on in the next session.

5.5. Study paperwork

Accurate completion of paperwork is a crucial part of research.

In the document titled ‘Enhanced upper limb therapy: participant goals and therapy record’ the following must be documented for each session:

- the therapy record which includes:
    - the goals worked on (record as D1, OG1, etc)
    - the number of repetitions of the task which have been practised for each goal. The total number of repetitions should be recorded.
    - the duration of the entire session
    - the duration of activity practice
    - the treating therapist/therapy assistant’s name and signature
    - the date
    - in the notes section any important aspects of therapy should be recorded. E.g. if a participant has achieved a part-task activity and should commence at the next part-task in the flowchart in the next therapy session, this should be recorded here.

In therapy sessions 11, 23 and 35, participant progress should also be documented:

- whether whole-task or part-task activities have been practised
- the starting and finishing position on the part-task flowchart (if appropriate)
- whether each goal has been achieved

© RATULS study team 2015

6. Senior therapist session summaries

6.1. Session 1: initial therapy session

[Total Duration: 60 minutes]

1. **Introduction** **[5 minutes]**
    - Description of RATULS enhanced therapy programme

2. **Assessment** **[15 minutes]**
    - Upper limb assessment
    - Goal setting and activity choice

3. **Warm-up stretching (if necessary)** **[2-5 minutes]**
    - Warm-up stretches and mobilisation of joints

4. **Core treatment** **[30 minutes]**

**Activity practice**
• Familiarise participant and therapy assistant with tasks<br>• Supervise activity practice

5. **Summary and feedback** **[5 minutes]**
    - Discuss participant's own evaluation of performance
    - Provide feedback

6. **Study paperwork** **[throughout session]**
    - Complete appropriate section of 'Enhanced upper limb therapy: Participant goal and therapy record' and, if relevant, 'Enhanced upper limb therapy: activity flowchart for optional goals or activity adaptions'.

© RATULS study team 2015

6.2. Sessions 12 and 24: Review sessions

[Total Duration: 60 minutes]

1. **Introduction** **[5 minutes]**
    - Review of participant progress

2. **Reassessment** **[15 minutes]**
    - Upper limb assessment
    - Goal setting and activity choice

3. **Warm-up stretching (if necessary)** **[2-5 minutes]**
    - Warm-up stretches and mobilisation of joints

4. **Core treatment** **[30 minutes]**

Activity practise

- Familiarise participant and therapy assistant with tasks
- Supervise activity practice

5. **Summary and feedback** **[5 minutes]**
    - Discuss participant's own evaluation of performance
    - Provide feedback

6. **Study paperwork** **[throughout session]**
    - Complete appropriate section of 'Enhanced upper limb therapy: Participant goal and therapy record' and, if relevant, 'Enhanced upper limb therapy: activity flowchart for optional goals or activity adaptions'.

© RATULS study team 2015

6.3. Session 36: Final therapy session

[Total Duration: 60 minutes]

1. Introduction **[5 minutes]**

- Review progress from previous session (session 35)
- Discuss activities to practise this session

2. Warm-up stretching (if necessary) **[2-5 minutes]**

- Warm-up stretches and mobilisation of joints

3. Core treatment **[30 minutes]**

**Activity practice**
Continue activity practice

4. Summary and feedback **[20 minutes]**

- Review goals and progress over entire programme
- Discuss participant's own evaluation of performance over entire programme
- Provide advice on longer term maintenance of upper limb function (e.g. home based exercises, local facilities)

5. Study paperwork **[throughout session]**

- Complete appropriate section of 'Enhanced upper limb therapy: Participant goal and therapy record'

© RATULS study team 2015

7. Therapy assistant session summaries

[Total Duration: 60 minutes]

1. Introduction [5 minutes]

- Review progress from previous session
- Discuss activities to practise this session

2. Warm-up stretching (if agreed at initial/review session) [2-5 minutes]

- Warm-up stretches and mobilisation of joints

3. Core treatment [45 minutes]

**Activity practice**
Continue activity practice

4. Summary and feedback [5 minutes]

- Discuss participant's own evaluation of performance
- Provide feedback

6. Study paperwork [throughout session]

- Complete appropriate section of 'Enhanced upper limb therapy: Participant goal and therapy record'.

Please note: for session 11, session 23 and session 35, participant progress should also be recorded. (In: 'Enhanced upper limb therapy: Participant goal and therapy record' section 'goal setting and review documentation', page 3)

© RATULS study team 2015

8. Recording therapy - participant goal and therapy record

Accurate completion of paperwork is a crucial part of research. The data recorded in the paperwork is necessary to describe the treatment participants receive during this therapy programme. This will be used to compare the dose of therapy received by participants in the enhanced therapy group and the robot-assisted training group.

Two documents are used to record the therapy programme:

1. Enhanced upper limb therapy: participant goal and therapy record.
2. Enhanced upper limb therapy: activity flowchart for optional goals or activity adaptations.

<u>Document 1: Enhanced upper limb therapy: participant goal and therapy record.</u>

Each participant will have a therapist-held participant goal and therapy record (figure 1).

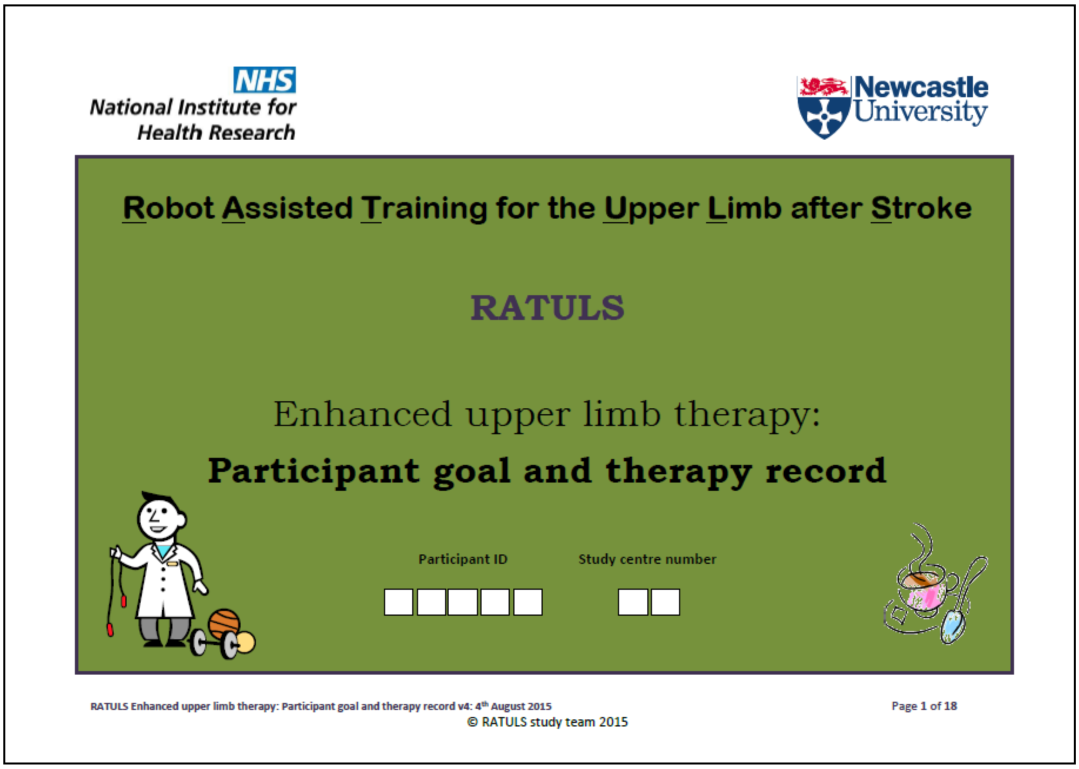

NHS
National Institute for Health Research

Newcastle University

Robot Assisted Training for the Upper Limb after Stroke

RATULS

Enhanced upper limb therapy:

Participant goal and therapy record

Participant ID

Study centre number

RATULS Enhanced upper limb therapy: Participant goal and therapy record v4: 4th August 2015

Page 1 of 18

© RATULS study team 2015

Figure 1: Front page of the participant goal and therapy record

© RATULS study team 2015

The following information should be entered at each session (figure 2):

- the session number
- the date
- the goals worked on (record as D1, OG1, etc)
- the number of repetitions of the task which have been practised for each goal
- the duration of the entire session
- the duration of activity practice
- the treating therapist/therapy assistant's name and signature

There is also a notes section which should be used to document important parts of a session which are not collected in the information described above. For example, if a participant has achieved a part-task activity and should commence at the next part-task in the flowchart in the next therapy session, this should be recorded here.

In addition, the participant ID should be added on each page.

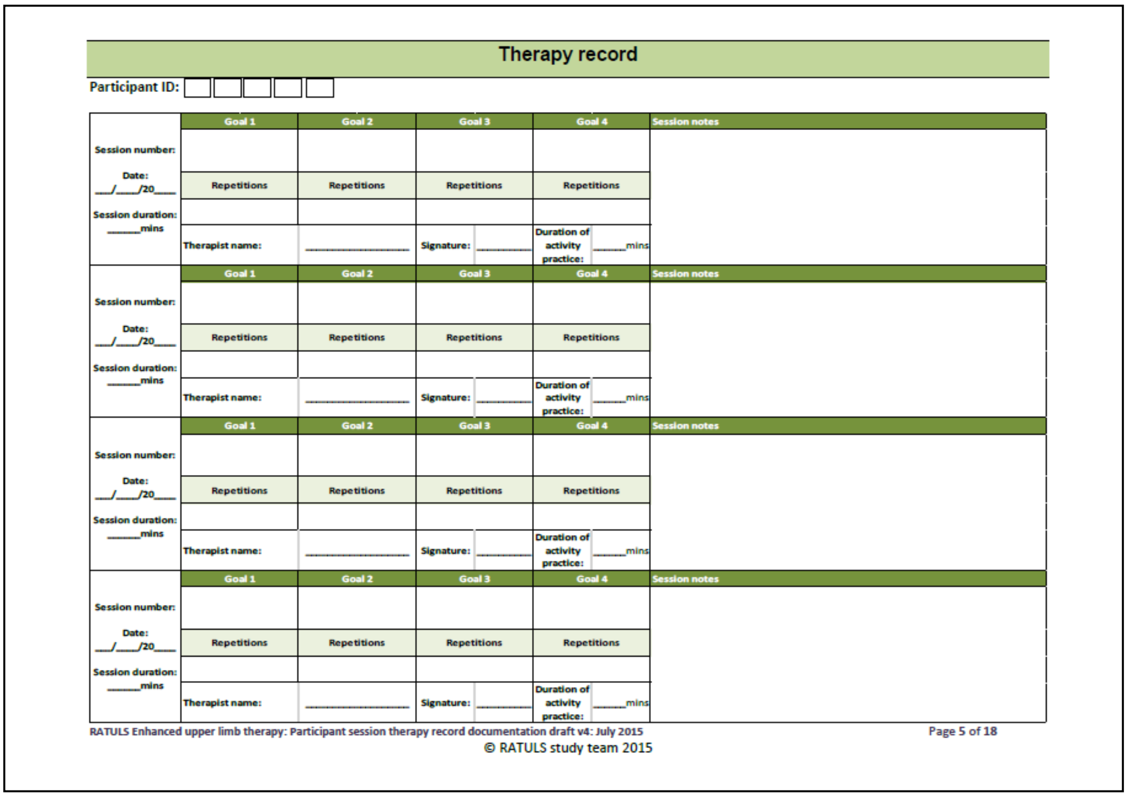

Therapy record

Participant ID: ☐☐☐☐☐

	Goal 1	Goal 2	Goal 3	Goal 4	Session notes
Session number:					
Date: ___/___/20___	Repetitions	Repetitions	Repetitions	Repetitions	
Session duration: ____mins					
	Therapist name:	________	Signature: ______	Duration of activity practice: ____mins	
	Goal 1	Goal 2	Goal 3	Goal 4	Session notes
Session number:					
Date: ___/___/20___	Repetitions	Repetitions	Repetitions	Repetitions	
Session duration: ____mins					
	Therapist name:	________	Signature: ______	Duration of activity practice: ____mins	
	Goal 1	Goal 2	Goal 3	Goal 4	Session notes
Session number:					
Date: ___/___/20___	Repetitions	Repetitions	Repetitions	Repetitions	
Session duration: ____mins					
	Therapist name:	________	Signature: ______	Duration of activity practice: ____mins	
	Goal 1	Goal 2	Goal 3	Goal 4	Session notes
Session number:					
Date: ___/___/20___	Repetitions	Repetitions	Repetitions	Repetitions	
Session duration: ____mins					
	Therapist name:	________	Signature: ______	Duration of activity practice: ____mins	

RATULS Enhanced upper limb therapy: Participant session therapy record documentation draft v4: July 2015 Page 5 of 18

© RATULS study team 2015

Figure 2: Example therapy session page of the participant goal and therapy record

An example of the completed paperwork is shown in figure 3.

© RATULS study team 2015

Therapy record

Participant ID: 5 3 4 1 4

	Goal 1	Goal 2	Goal 3	Goal 4	Session notes
Session number: 1	W2	F1	OG1 - Using a knife	PC1 Brushing hair	For goal F1 practise progressed from position F1.03 to F1.04
Date: 04/08/2015	Repetitions	Repetitions	Repetitions	Repetitions	
Session duration: 60 mins	95	85	78	102	
	Therapist name: HELEN BOSOMWORTH		Signature: [illegible]	Duration of activity practice: 45 mins	

	Goal 1	Goal 2	Goal 3	Goal 4	Session notes
Session number:					
Date: ___/___/20___	Repetitions	Repetitions	Repetitions	Repetitions	
Session duration: ______mins					
	Therapist name: ________		Signature: ________	Duration of activity practice: ______mins	

	Goal 1	Goal 2	Goal 3	Goal 4	Session notes
Session number:					
Date: ___/___/20___	Repetitions	Repetitions	Repetitions	Repetitions	
Session duration: ______mins					
	Therapist name: ________		Signature: ________	Duration of activity practice: ______mins	

	Goal 1	Goal 2	Goal 3	Goal 4	Session notes
Session number:					
Date: ___/___/20___	Repetitions	Repetitions	Repetitions	Repetitions	
Session duration: ______mins					
	Therapist name: ________		Signature: ________	Duration of activity practice: ______mins	

RATULS Enhanced upper limb therapy: Participant session therapy record documentation draft v4: July 2015 Page 4 of 18

© RATULS study team 2015

Figure 3: Example of a completed therapy record for one session

© RATULS study team 2015

At the initial therapy session and sessions 12 and 24, the upper limb assessment can be documented (figure 4): This is located on pages 16, 17 and 18 of the document.

Upper limb assessment

Upper limb assessment to be completed by senior therapist

Session number: _____
Side of body affected by current stroke: _______________

Prompts:

- Selective movement
- Passive range of movement
- Muscle tone
- Compensations
- Proprioception
- Associated reactions
- Pain

**Shoulder**		**Wrist**	
**Elbow**		**Hand**	

Inattention / other comments:
____
____
____

RATULS Enhanced upper limb therapy: Participant goal and therapy record v4: 4th August 2015 Page 17 of 18
© RATULS study team 2015

Figure 4: Example of the upper limb assessment record.

Goal choices and selection of whole-task/part-task practice plus goal attainment are recorded on page 3 ‘goal setting and review documentation’ section (figure 5).

© RATULS study team 2015

Goal setting and review documentation

Participant ID: ☐☐☐☐☐

	Goal 1				Goal 2				Goal 3				Goal 4			
Session 1																
Week 1 Initial session	Whole (tick)	Part (tick)	Current Position	Part of task (? / ?)	Whole (tick)	Part (tick)	Current Position	Part of task (? / ?)	Whole (tick)	Part (tick)	Current Position	Part of task (? / ?)	Whole (tick)	Part (tick)	Current Position	Part of task (? / ?)
Session number:	Goal 1				Goal 2				Goal 3				Goal 4			
	Current Position		Part of task (? / ?)	Achieved (Y/N)	Current Position		Part of task (? / ?)	Achieved (Y/N)	Current Position		Part of task (? / ?)	Achieved (Y/N)	Current Position		Part of task (? / ?)	Achieved (Y/N)
Progress since week 1																
	Goal 1				Goal 2				Goal 3				Goal 4			
Session number:	Week 1 Goal 1 Achieved (Y/N)				Week 1 Goal 2 Achieved (Y/N)				Week 1 Goal 3 Achieved (Y/N)				Week 1 Goal 4 Achieved (Y/N)			
Week 4 Review session	Whole (tick)	Part (tick)	Current Position	Part of task (? / ?)	Whole (tick)	Part (tick)	Current Position	Part of task (? / ?)	Whole (tick)	Part (tick)	Current Position	Part of task (? / ?)	Whole (tick)	Part (tick)	Current Position	Part of task (? / ?)
Session number:	Goal 1				Goal 2				Goal 3				Goal 4			
	Current Position		Part of task (? / ?)	Achieved (Y/N)	Current Position		Part of task (? / ?)	Achieved (Y/N)	Current Position		Part of task (? / ?)	Achieved (Y/N)	Current Position		Part of task (? / ?)	Achieved (Y/N)
Progress since week 4																
	Goal 1				Goal 2				Goal 3				Goal 4			
Session number:	Week 4 Goal 1 Achieved (Y/N)				Week 4 Goal 2 Achieved (Y/N)				Week 4 Goal 3 Achieved (Y/N)				Week 4 Goal 4 Achieved (Y/N)			
Week 8 Review session	Whole (tick)	Part (tick)	Current Position	Part of task (? / ?)	Whole (tick)	Part (tick)	Current Position	Part of task (? / ?)	Whole (tick)	Part (tick)	Current Position	Part of task (? / ?)	Whole (tick)	Part (tick)	Current Position	Part of task (? / ?)
Session number:	Goal 1				Goal 2				Goal 3				Goal 4			
	Current Position		Part of task (? / ?)	Achieved (Y/N)	Current Position		Part of task (? / ?)	Achieved (Y/N)	Current Position		Part of task (? / ?)	Achieved (Y/N)	Current Position		Part of task (? / ?)	Achieved (Y/N)
Progress since week 8																
Final session	Week 8 Goal 1 Achieved (Y/N)				Week 8 Goal 2 Achieved (Y/N)				Week 8 Goal 3 Achieved (Y/N)				Week 8 Goal 4 Achieved (Y/N)			

RATULS Enhanced upper limb therapy: Participant goal and therapy record v4: 4th August 2015 Page 3 of 18

© RATULS study team 2015

Figure 5: Example of the goal setting and review documentation.

© RATULS study team 2015

At the initial session, and sessions 12 and 24 (week 4 and week 8 review respectively), the goal choice and type of activity practice should be recorded. The following should be documented:

- the (up to four) goals that have been selected
    - if a goal has been selected from the pre-specified list, please document this by its number as listed in 'Enhanced upper limb therapy 3: warm up stretching, goal choices and activity flowcharts' (e.g. D1).
    - If an alternative goal has been chosen ('optional goal'), this should be written out free text and labelled as 'OG 1' (optional goal 1). If more than one optional goal has been chosen these should also be written out and then labelled as OG 2, OG 3, etc. For example: 'Using a knife and fork – OG1'.
- whether whole-task of part-task activity practice has been chosen, for each goal.
- where part-task practice has been chosen for a pre-specified goal, the start position on the relevant flowchart should be recorded (indicated by the number in the flowchart box e.g. W1.02)
- for part task practice it is optional to record how many parts of a task exist and where the participant should begin on that task. E.g. if a task has 5 parts and the participant should begin on part 2 then for the initial session 'part of task' is 2/5.

An example of the completed paperwork is shown in figure 6.

© RATULS study team 2015

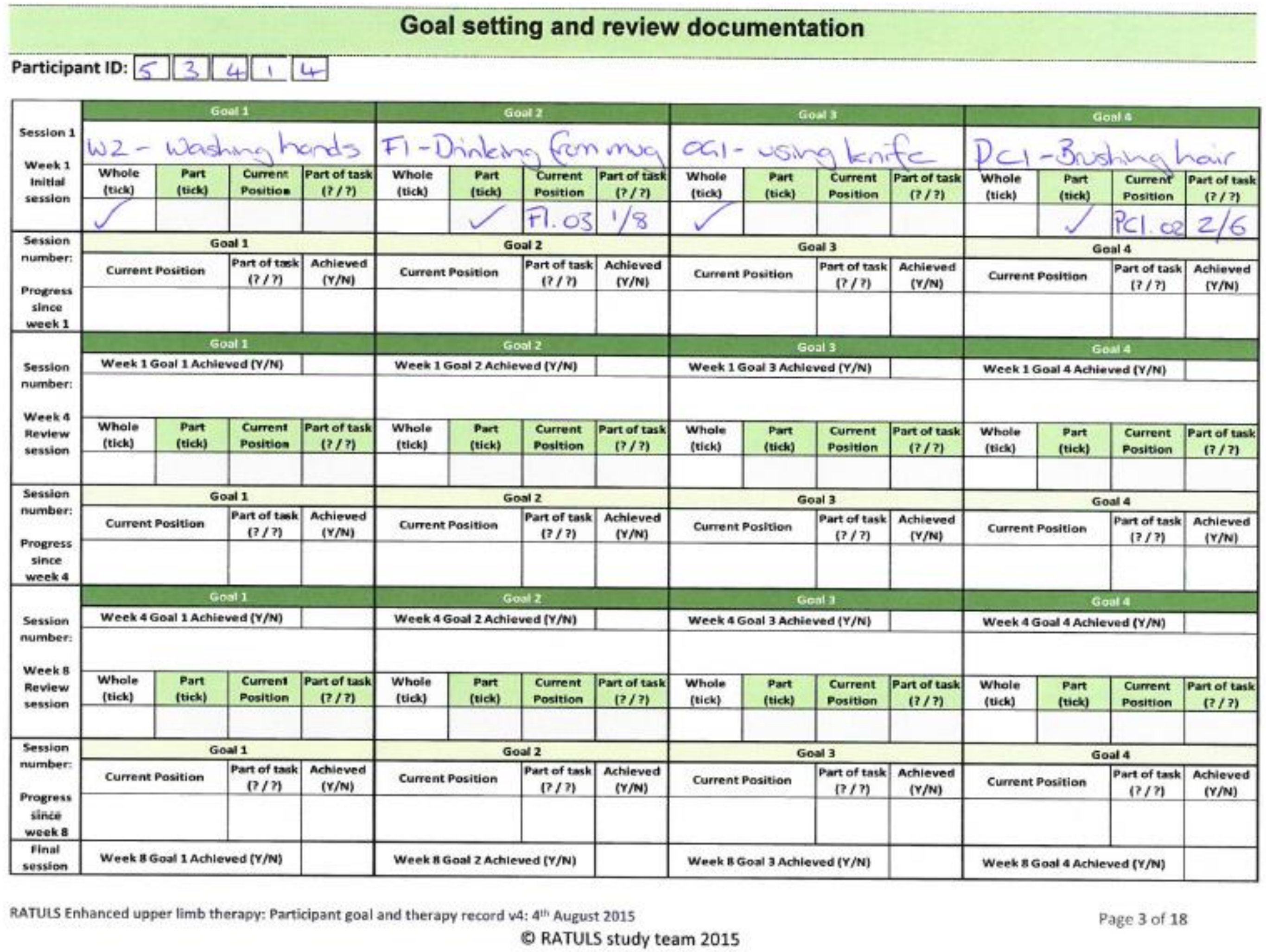

Goal setting and review documentation

Participant ID: 5 3 4 1 4

Session 1 Week 1 Initial session	Goal 1				Goal 2				Goal 3				Goal 4			
	W2 - Washing hands				F1 - Drinking from mug				OG1 - using knife				PC1 - Brushing hair			
	Whole (tick)	Part (tick)	Current Position	Part of task (? / ?)	Whole (tick)	Part (tick)	Current Position	Part of task (? / ?)	Whole (tick)	Part (tick)	Current Position	Part of task (? / ?)	Whole (tick)	Part (tick)	Current Position	Part of task (? / ?)
	✓					✓	F1.03	1/8	✓					✓	PC1.02	2/6

Session number: Progress since week 1	Goal 1			Goal 2			Goal 3			Goal 4		
	Current Position	Part of task (? / ?)	Achieved (Y/N)	Current Position	Part of task (? / ?)	Achieved (Y/N)	Current Position	Part of task (? / ?)	Achieved (Y/N)	Current Position	Part of task (? / ?)	Achieved (Y/N)

Session number: Week 4 Review session	Goal 1				Goal 2				Goal 3				Goal 4			
	Week 1 Goal 1 Achieved (Y/N)				Week 1 Goal 2 Achieved (Y/N)				Week 1 Goal 3 Achieved (Y/N)				Week 1 Goal 4 Achieved (Y/N)			
	Whole (tick)	Part (tick)	Current Position	Part of task (? / ?)	Whole (tick)	Part (tick)	Current Position	Part of task (? / ?)	Whole (tick)	Part (tick)	Current Position	Part of task (? / ?)	Whole (tick)	Part (tick)	Current Position	Part of task (? / ?)

Session number: Progress since week 4	Goal 1			Goal 2			Goal 3			Goal 4		
	Current Position	Part of task (? / ?)	Achieved (Y/N)	Current Position	Part of task (? / ?)	Achieved (Y/N)	Current Position	Part of task (? / ?)	Achieved (Y/N)	Current Position	Part of task (? / ?)	Achieved (Y/N)

Session number: Week 8 Review session	Goal 1				Goal 2				Goal 3				Goal 4			
	Week 4 Goal 1 Achieved (Y/N)				Week 4 Goal 2 Achieved (Y/N)				Week 4 Goal 3 Achieved (Y/N)				Week 4 Goal 4 Achieved (Y/N)			
	Whole (tick)	Part (tick)	Current Position	Part of task (? / ?)	Whole (tick)	Part (tick)	Current Position	Part of task (? / ?)	Whole (tick)	Part (tick)	Current Position	Part of task (? / ?)	Whole (tick)	Part (tick)	Current Position	Part of task (? / ?)

Session number: Progress since week 8	Goal 1			Goal 2			Goal 3			Goal 4		
	Current Position	Part of task (? / ?)	Achieved (Y/N)	Current Position	Part of task (? / ?)	Achieved (Y/N)	Current Position	Part of task (? / ?)	Achieved (Y/N)	Current Position	Part of task (? / ?)	Achieved (Y/N)
Final session	Week 8 Goal 1 Achieved (Y/N)			Week 8 Goal 2 Achieved (Y/N)			Week 8 Goal 3 Achieved (Y/N)			Week 8 Goal 4 Achieved (Y/N)		

RATULS Enhanced upper limb therapy: Participant goal and therapy record v4: 4th August 2015 Page 3 of 18

© RATULS study team 2015

Figure 6: Example session 1 completed goal setting and review documentation.

© RATULS study team 2015

At sessions 11, 23, and 35, participant progress should be recorded to assist with the next review session. This is progress since week 1, progress since week 4 and progress since week 8 respectively. The following should be documented:

- where part-task practice was chosen for a pre-specified goal, the current position on the relevant flowchart should be recorded (indicated by the number in the flowchart box e.g. W1.02)
- for part task practice It is optional to record how many parts of a task have been completed. E.g. if a task has 5 parts and the participant has begun on part 2 (as specified in their goal setting session) but has progressed to part 4 then 'part of task' is 4/5.
- whether each goal has been achieved

An example of the completed paperwork is shown in figure 7.

At the review sessions (12 and 24), in addition to the goal setting information described above (page 29), achievement of goals from the previous four weeks should be recorded. An example of the completed paperwork is shown in figure 8.

© RATULS study team 2015

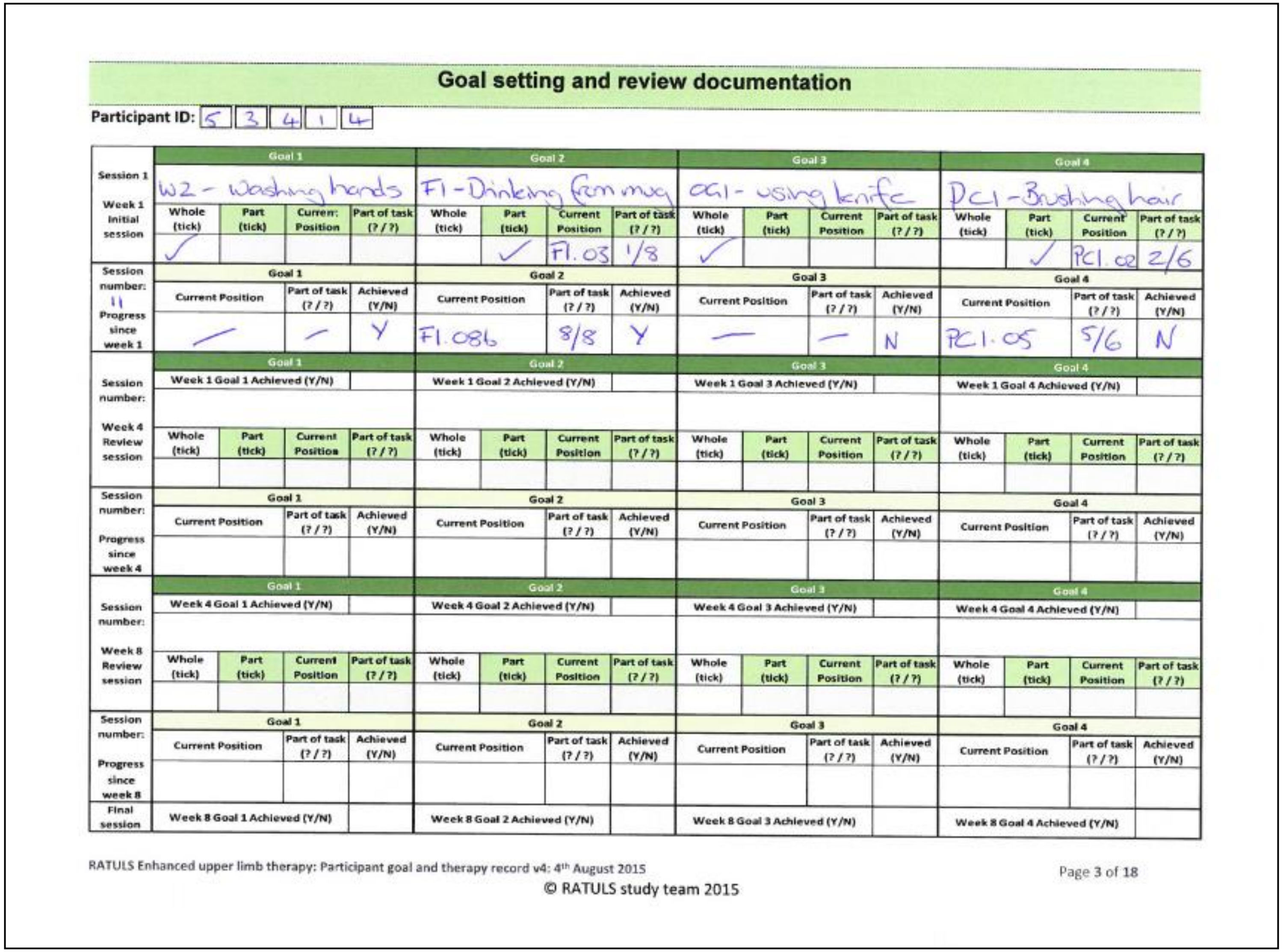

Goal setting and review documentation

Participant ID: 5 3 4 1 4

	Goal 1				Goal 2				Goal 3				Goal 4			
Session 1 Week 1 Initial session	W2 - Washing hands				F1 - Drinking from mug				OG1 - using knife				PC1 - Brushing hair			
	Whole (tick)	Part (tick)	Current Position	Part of task (? / ?)	Whole (tick)	Part (tick)	Current Position	Part of task (? / ?)	Whole (tick)	Part (tick)	Current Position	Part of task (? / ?)	Whole (tick)	Part (tick)	Current Position	Part of task (? / ?)
	✓					✓	F1.03	1/8	✓					✓	PC1.02	2/6
Session number: 11 Progress since week 1	Current Position		Part of task (? / ?)	Achieved (Y/N)	Current Position		Part of task (? / ?)	Achieved (Y/N)	Current Position		Part of task (? / ?)	Achieved (Y/N)	Current Position		Part of task (? / ?)	Achieved (Y/N)
	—		—	Y	F1.086		8/8	Y	—		—	N	PC1.05		5/6	N
Session number: Week 4 Review session	Week 1 Goal 1 Achieved (Y/N)				Week 1 Goal 2 Achieved (Y/N)				Week 1 Goal 3 Achieved (Y/N)				Week 1 Goal 4 Achieved (Y/N)			
	Whole (tick)	Part (tick)	Current Position	Part of task (? / ?)	Whole (tick)	Part (tick)	Current Position	Part of task (? / ?)	Whole (tick)	Part (tick)	Current Position	Part of task (? / ?)	Whole (tick)	Part (tick)	Current Position	Part of task (? / ?)
Session number: Progress since week 4	Current Position		Part of task (? / ?)	Achieved (Y/N)	Current Position		Part of task (? / ?)	Achieved (Y/N)	Current Position		Part of task (? / ?)	Achieved (Y/N)	Current Position		Part of task (? / ?)	Achieved (Y/N)
Session number: Week 8 Review session	Week 4 Goal 1 Achieved (Y/N)				Week 4 Goal 2 Achieved (Y/N)				Week 4 Goal 3 Achieved (Y/N)				Week 4 Goal 4 Achieved (Y/N)			
	Whole (tick)	Part (tick)	Current Position	Part of task (? / ?)	Whole (tick)	Part (tick)	Current Position	Part of task (? / ?)	Whole (tick)	Part (tick)	Current Position	Part of task (? / ?)	Whole (tick)	Part (tick)	Current Position	Part of task (? / ?)
Session number: Progress since week 8	Current Position		Part of task (? / ?)	Achieved (Y/N)	Current Position		Part of task (? / ?)	Achieved (Y/N)	Current Position		Part of task (? / ?)	Achieved (Y/N)	Current Position		Part of task (? / ?)	Achieved (Y/N)
Final session	Week 8 Goal 1 Achieved (Y/N)				Week 8 Goal 2 Achieved (Y/N)				Week 8 Goal 3 Achieved (Y/N)				Week 8 Goal 4 Achieved (Y/N)			

RATULS Enhanced upper limb therapy: Participant goal and therapy record v4: 4th August 2015 Page 3 of 18

© RATULS study team 2015

Figure 7: Example session 11 (progress since week 1) completed goal setting and review documentation.

© RATULS study team 2015

Goal setting and review documentation

Participant ID: 5 3 4 1 4

	Goal 1				Goal 2				Goal 3				Goal 4			
Session 1 Week 1 Initial session	W2 - Washing hands				F1 - Drinking from mug				OG1 - using knife				PC1 - Brushing hair			
	Whole (tick)	Part (tick)	Current Position	Part of task (?/?)	Whole (tick)	Part (tick)	Current Position	Part of task (?/?)	Whole (tick)	Part (tick)	Current Position	Part of task (?/?)	Whole (tick)	Part (tick)	Current Position	Part of task (?/?)
	✓					✓	F1.03	1/8	✓					✓	PC1.02	2/6
Session number: 11 Progress since week 1	Current Position		Part of task (?/?)	Achieved (Y/N)	Current Position		Part of task (?/?)	Achieved (Y/N)	Current Position		Part of task (?/?)	Achieved (Y/N)	Current Position		Part of task (?/?)	Achieved (Y/N)
	—		—	Y	F1.086		8/8	Y	—		—	N	PC1.05		5/6	N
Session number: 12 Week 4 Review session	Week 1 Goal 1 Achieved (Y/N)				Week 1 Goal 2 Achieved (Y/N)				Week 1 Goal 3 Achieved (Y/N)				Week 1 Goal 4 Achieved (Y/N)			
	OG2 - Washing dishes				D1 - Closing a zip				OG1 - using knife				PC1 - Brushing hair			
	Whole (tick)	Part (tick)	Current Position	Part of task (?/?)	Whole (tick)	Part (tick)	Current Position	Part of task (?/?)	Whole (tick)	Part (tick)	Current Position	Part of task (?/?)	Whole (tick)	Part (tick)	Current Position	Part of task (?/?)
	✓				✓				✓					✓	PC1.05	5/6
Session number: Progress since week 4	Current Position		Part of task (?/?)	Achieved (Y/N)	Current Position		Part of task (?/?)	Achieved (Y/N)	Current Position		Part of task (?/?)	Achieved (Y/N)	Current Position		Part of task (?/?)	Achieved (Y/N)
Session number: Week 8 Review session	Week 4 Goal 1 Achieved (Y/N)				Week 4 Goal 2 Achieved (Y/N)				Week 4 Goal 3 Achieved (Y/N)				Week 4 Goal 4 Achieved (Y/N)			
	Whole (tick)	Part (tick)	Current Position	Part of task (?/?)	Whole (tick)	Part (tick)	Current Position	Part of task (?/?)	Whole (tick)	Part (tick)	Current Position	Part of task (?/?)	Whole (tick)	Part (tick)	Current Position	Part of task (?/?)
Session number: Progress since week 8	Current Position		Part of task (?/?)	Achieved (Y/N)	Current Position		Part of task (?/?)	Achieved (Y/N)	Current Position		Part of task (?/?)	Achieved (Y/N)	Current Position		Part of task (?/?)	Achieved (Y/N)
Final session	Week 8 Goal 1 Achieved (Y/N)				Week 8 Goal 2 Achieved (Y/N)				Week 8 Goal 3 Achieved (Y/N)				Week 8 Goal 4 Achieved (Y/N)			

RATULS Enhanced upper limb therapy: Participant goal and therapy record v4: 4th August 2015 Page 3 of 18

© RATULS study team 2015

Figure 8: Example session 12 (week 4 review session) completed goal setting and review documentation.

© RATULS study team 2015

At session 36, achievement of goals should be recorded on the goal setting and review documentation, the therapy record should be completed and the senior therapist is required to give feedback to the participant about their progress over the enhanced upper limb therapy programme. Further advice should also be given regarding maintaining upper limb function (e.g. home exercises). This should be documented (figure 9).

Final session feedback and advice

Participant ID: ☐☐☐☐☐

Feedback given to participant about progress over the programme

Further advice given regarding maintaining upper limb function (e.g home exercises)

RATULS Enhanced upper limb therapy: Participant goal and therapy record v4: 4th August 2015 Page 13 of 18

© RATULS study team 2015

Figure 9: Example of the final session feedback and advice documentation.

© RATULS study team 2015

Document 2: Enhanced upper limb therapy: activity flowchart for optional goals or activity adaptations

The blank activity flowcharts (see figure 10) should be used to document activities to practice for optional goals and can be used to show adaptations to the pre-specified activity flowcharts. In addition, there are spaces to include any further information or guidance that should be followed in the therapy sessions.

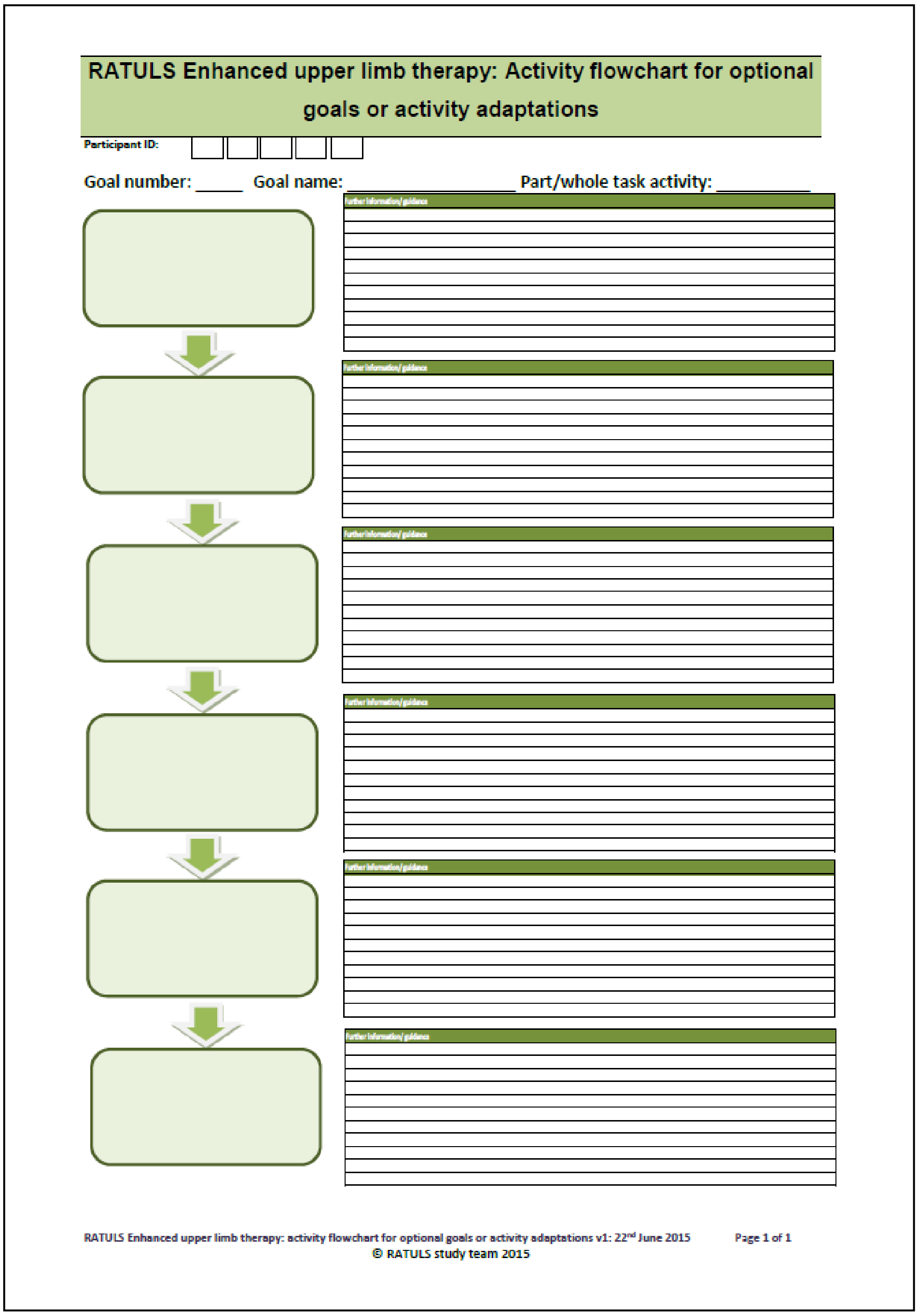

RATULS Enhanced upper limb therapy: Activity flowchart for optional goals or activity adaptations

Participant ID:

Goal number: _____ Goal name: ________________ Part/whole task activity: __________

RATULS Enhanced upper limb therapy: activity flowchart for optional goals or activity adaptations v1: 22nd June 2015 Page 1 of 1

© RATULS study team 2015

Figure 10: Example of an activity flowchart for optional goals or activity adaptations.

© RATULS study team 2015

9. Participant attendance

The RATULS enhanced upper limb therapy programme is designed to be followed session by session. However, it is inevitable that some participants will be unavailable for some sessions.

If a participant misses a session, this can be 'made up' according to local capacity. For example, if a participant was due to attend session 10 on Wednesday but was unavailable, if capacity allowed, the participant could attend for session 10 on Thursday instead.

If capacity does not allow a session to be 'made up', the session is lost. If a session is lost, this should be documented using a 'did not attend record' (see figure 11).

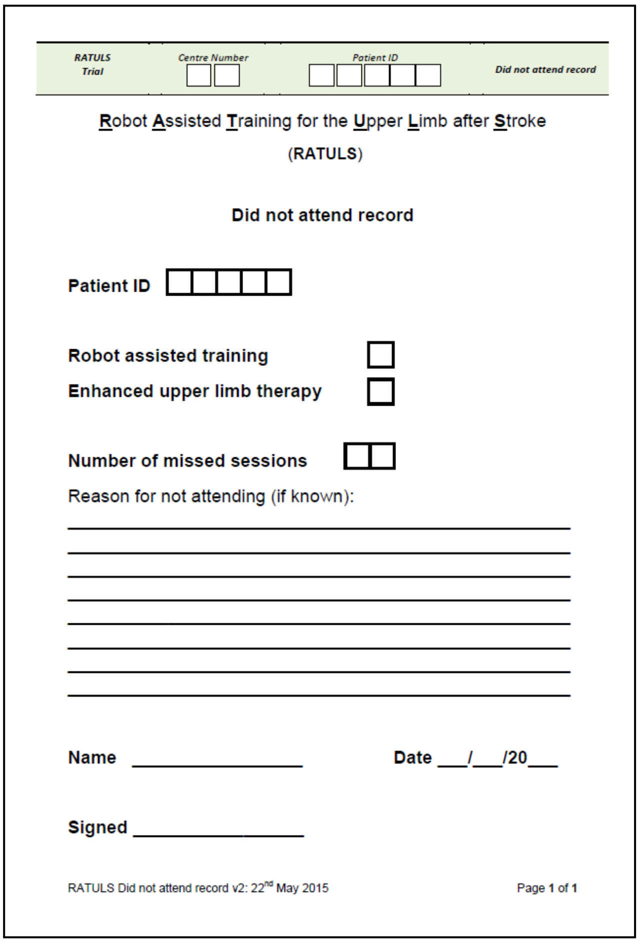

RATULS Trial | Centre Number ☐☐ | Patient ID ☐☐☐☐☐ | Did not attend record

Robot Assisted Training for the Upper Limb after Stroke

(RATULS)

Did not attend record

Patient ID ☐☐☐☐☐

Robot assisted training ☐

Enhanced upper limb therapy ☐

Number of missed sessions ☐☐

Reason for not attending (if known):

____

____

____

____

____

____

____

____

Name ________________ **Date** ___/___/20___

Signed ________________

RATULS Did not attend record v2: 22nd May 2015 Page 1 of 1

Figure 11: Example of a did not attend record.

© RATULS study team 2015

The only exception to this guidance is when a participant is unavailable to attend a scheduled ‘review’ session (session 12 or 24). In this case, if this session cannot be ‘made up’, the review session should be held when the participant can next attend and a later therapy session should be lost. This is because it will be necessary to conduct the review sessions to progress treatment.

© RATULS study team 2015

10. Enhanced upper limb therapy documents

This Enhanced upper limb therapy 2: How to deliver the programme manual is part of a series of documents developed to describe the RATULS enhanced therapy programme. The other documents in this series are:

- Enhanced upper limb therapy 1: Programme overview
- Enhanced upper limb therapy 3: Warm-up stretches, goal choices and activity flowcharts

11. RATULS co-ordinating centre

For further information please contact the RATULS co-ordinating team:

Stroke Research Group

3-4 Claremont Terrace

Newcastle University

Newcastle Upon Tyne

NE2 4AE

Email: ratuls@ncl.ac.uk

Phone: 0191 2086779

Or refer to the RATULS website: http://research.ncl.ac.uk/ratuls/

12. References

1. Birkenmeier, R.L., E.M. Prager, and C.E. Lang, Neurorehabil Neural Repair, Translating animal doses of task-specific training to people with chronic stroke in 1-hour therapy sessions: a proof-of-concept study. 2010. **24**(7): p. 620-35.

© RATULS study team 2015

This enhanced upper limb therapy manual has been produced by Dr Helen Bosomworth with input from Miss Lianne Brkic, Mrs Lydia Aird, Dr Lisa Shaw and Professor Frederike van Wijck.

© RATULS study team 2015:
Helen Rodgers, Newcastle University
Lydia Aird, Northumbria Healthcare NHS Foundation Trust
Sreeman Andole, Barking, Havering & Redbridge University Hospitals NHS Trust
David Cohen, London North West Hospitals NHS Trust
Jesse Dawson, University of Glasgow
Janet Eyre, Newcastle University
Tracy Finch, Newcastle University
Gary Ford, Newcastle University
Steven Hogg, lay member
Denise Howel, Newcastle University
Niall Hughes, NHS Greater Glasgow and Clyde
Hermano Igo Krebs, Massachusetts Institute of Technology
Chris Price, Northumbria Healthcare NHS Foundation Trust
Lynn Rochester, Newcastle University
Lisa Shaw, Newcastle University
Laura Ternent, Newcastle University
Duncan Turner, University of East London
Luke Vale, Newcastle University
Frederike van Wijck, Glasgow Caledonian University
Scott Wilkes, University of Sunderland

© RATULS study team 2015

National Institute for Health Research

Newcastle University

Robot Assisted Training for the Upper Limb after Stroke

RATULS

Enhanced upper limb therapy 3: **Warm-up stretches, goal choices and activity flowcharts**

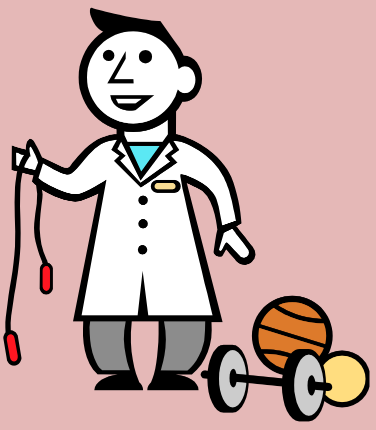

© RATULS study team 2015

Contents

1. Purpose of this document 3

2. Warm-up stretches 3

3. Upper limb rehabilitation goal choices 6
   Washing (W) 6
   Dressing (D) 6
   Eating and Drinking (F) 6
   Other 6
   Optional goals 7

4. Activity Flowcharts 8
Washing (W) 9
   W1 – Using a sponge/ flannel 9
   W2 – Washing hands/ face/ hair 11
   W3 – Cleaning Teeth 13
   W4 – Hand care 15
Dressing (D) 17
   D1 – Closing a zip/ buttons 17
   D2 – Putting on a cardigan/ coat/ shirt 19
   D3 – Putting on a mitten/ glove 21
   D4 – Putting on socks/ shoes 23
   D5 – Putting on spectacles 25
Eating and Drinking (F) 27
   F1 – Drinking from mug/ glass 27
   F2 – Pouring from bottle/ jug/ kettle 29
   F3 – Removing lids/ open containers 31
Other 33
   PC1 – Brushing hair 33
   FM1 – Opening doors with affected hand 35
   FM2 – Sit-to-stand using both hands 37

5. Enhanced upper limb therapy documents 40

6. RATULS co-ordinating centre 40

© RATULS study team 2015

1. Purpose of this document

This document describes example warm-up stretches, lists goal choices and provides activity flowcharts for the RATULS enhanced upper limb therapy programme. It is intended to serve as a reference and resource guide for clinicians delivering the enhanced upper limb therapy programme.

2. Warm-up stretches

Prior to practising activities in the RATULS enhanced upper limb therapy programme, gentle warm up stretching can be performed where necessary. A "warm-up" helps to focus attention on the affected arm, and stimulate sensation and proprioception by gently stretching soft tissues and mobilising the joints, before practising the rehabilitation activities. However, warm-up stretches should be kept to a minimum as the focus of the sessions should be on task specific activity practice.

The following stretches are given as a guide. Different stretches may be used if advised by a senior therapist. The therapy assistants must follow advice of the senior therapist.

© RATULS study team 2015

Stretch 1 – this stretches down the full length of the participants arm.

1. Participant to place the affected hand flat on the table (palm down), with assistance from the therapist/ therapy assistant where necessary.
2. Therapist/ therapy assistant to take hold of the wrist of the affected arm.
3. Slowly slide the affected hand forwards on the table so that the elbow straightens out. The participant should not feel any pain.
4. Make sure the body remains still and the participant doesn't lean forward. Then relax.
5. Hold for approximately 20 – 30 seconds. Then relax.
6. Repeat as appropriate.

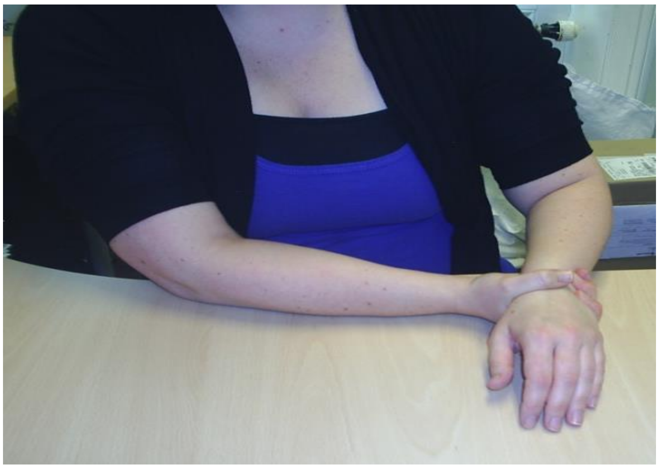

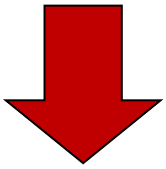

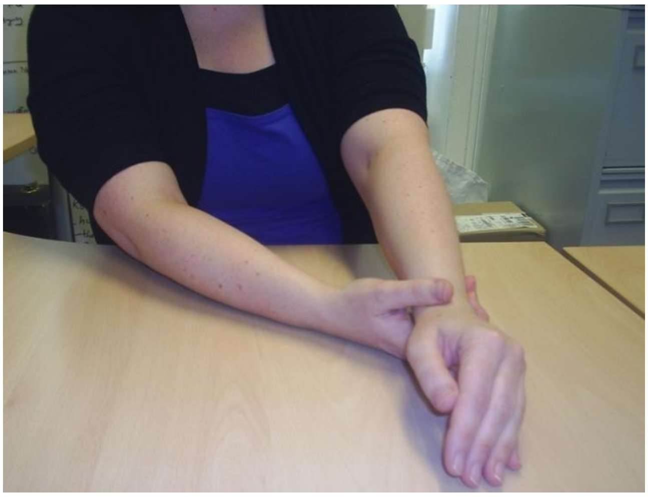

© RATULS study team 2015

Stretch 2 - focuses on the participant's wrist and fingers

1. Participant to place the hand flat on the table in front with assistance from the therapist/ therapy assistant where necessary. Ensure the thumb is in abduction.
2. Therapist/ therapy assistant to slide the fingers of the unaffected hand under the fingers of the affected hand.
3. Make sure the participant's wrist stays on the table.
4. Slowly lift the participant's fingers up (keeping them straight). The aim is to bend the wrist backwards.
5. The participant should feel a gentle stretch down their fingers, palm and wrist. The participant should not feel any pain.
6. Hold for approximately 20 – 30 seconds. Then relax.
7. Repeat as appropriate.

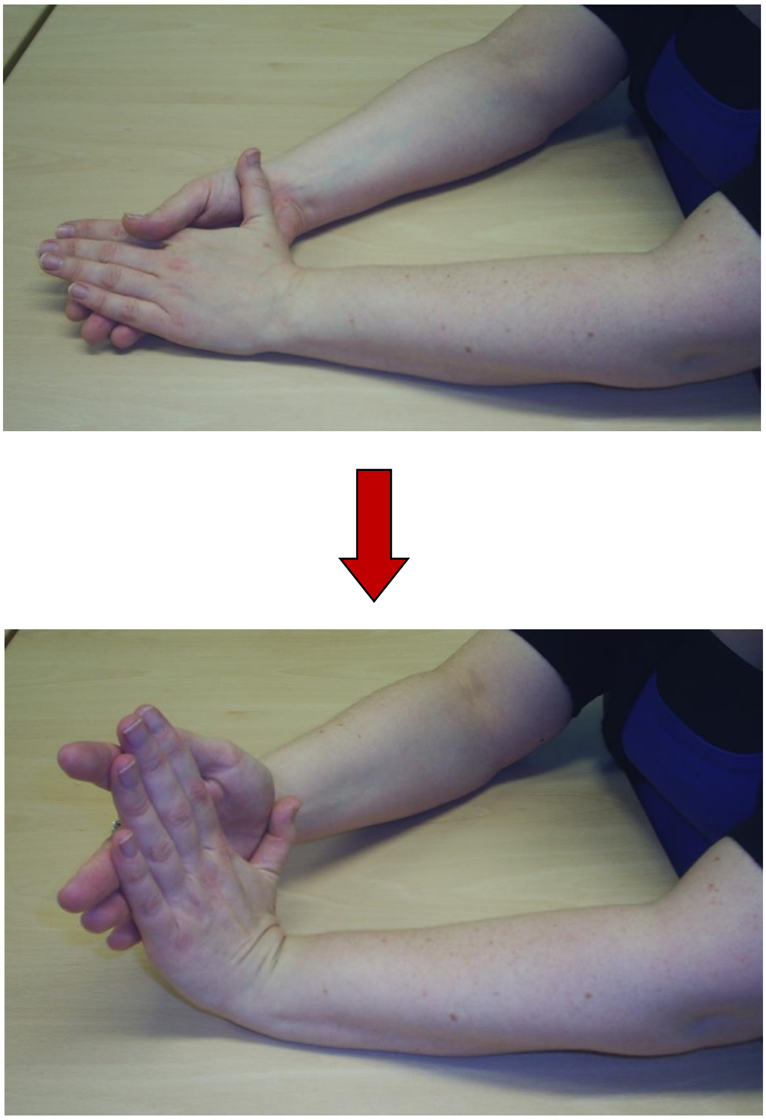

© RATULS study team 2015

3. Upper limb rehabilitation goal choices

The following rehabilitation goals are suggestions for use in the RATULS enhanced upper limb therapy programme.

Washing (W)

W1. Using a sponge/ flannel
W2. Washing hands/ face/ hair
W3. Cleaning teeth
W4. Hand care

Dressing (D)

D1. Closing a zip/ buttons
D2. Putting on a cardigan/ coat/ shirt
D3. Putting on a mitten/ glove
D4. Putting on socks/ shoes
D5. Putting on spectacles

Eating and Drinking (F)

F1. Drinking from mug/ glass
F2. Pouring from a bottle/ jug/ kettle
F3. Removing lids/ open containers

Other

Personal Care

PC1. Brushing hair

Functional Mobility

FM1. Opening doors with affected hand
FM2. Sit-to-stand using both hands

© RATULS study team 2015

Optional goals

Whilst it is anticipated that the goal choices listed above will cover many participant choices, it is acceptable for alternative goals to be set at local discretion by the senior therapist.

If an alternative goal has been chosen ('optional goal'), this goal should be written out free text into the 'Enhanced upper limb therapy: participant goal and therapy record and labelled as 'OG 1' (optional goal 1). If more than one optional goal has been chosen these should also be written out and then labelled as OG 2, OG 3, etc.

For example: 'Using a knife and fork – OG1'.

© RATULS study team 2015

4. Activity Flowcharts

For each goal choice, two activity flowcharts are available. The first flowchart show a 'whole-task' activity. The second flowchart, 'part-task' activities.

© RATULS study team 2015

Washing (W)

W1 – Using a sponge/ flannel

1. Whole-task activity:

Sitting on a perching stool/seat

NO GRASP

- Place affected hand in supported position on the sink.
- Encourage abduction at shoulder to wash under affected arm. (Possibly hand over hand if some shoulder/arm function.)
- Wash affected arm – encouraging maintained position.
- Repeat the process.

SOME GRASP

- Place sponge in affected hand or reach for it if possible. (Assist with unaffected arm or therapist to facilitate if required.)
- Take hand to appropriate body part e.g. face. (May require support at the elbow.)
- Assist from elbow to move sponge around face. (May require some assistance at the hand.)
- Take hand/sponge down into the water. (Assist where required.)
- Bimanual wringing of sponge where possible.
- Place sponge down and release.
- Repeat the process.

Possible progression:

- Introduce soap/shower gel
- Reduce the amount of verbal cues
- Reduce the amount of facilitation

© RATULS study team 2015

2. Part-task activities:

Sitting on a perching stool/seat

W1.01 Move affected hand from lap to the table and back to lap.

W1.02 Reach affected hand to sponge/ flannel and return to lap.

NO GRASP

Assist with unaffected arm or therapist to facilitate if required

W1.03a Reach affected hand to the sink and place hand into a supported position.

W1.04a Reach affected hand and arm into supported position and encourage abduction at the shoulder.

W1.05a With arm and hand in supported position abduct shoulder and wash affected arm with unaffected hand.

SOME GRASP

W1.03b Facilitate fingers of affected hand around sponge/ flannel then release.

Assist with unaffected arm or therapist to facilitate if required

W1.04b Reach for sponge/flannel with affected hand and take to unaffected hand and back.

May require help with maintaining position if there is weakness at the elbow and/or shoulder.

W1.05b Reach for sponge/flannel with affected hand and take to unaffected elbow and back.

W1.06b Reach for sponge/flannel with affected hand and take to unaffected shoulder and back.

W1.07b Reach for sponge/flannel with affected hand and wash unaffected. Release the sponge/flannel.

GOAL ACHIEVED

Possible progression:

- Introduce soap/shower gel
- Reduce the amount of verbal cues
- Reduce the amount of facilitation

© RATULS study team 2015

W2 – Washing hands/ face/ hair

1. Whole-task activity:

Sitting on a perching stool/seat

Place affected hand in bowl assisting with unaffected.

May need assistance to stabilise hand in the bowl.

Put soap in affected hand and lather with unaffected.

As an alternative use a soaped flannel if the soap too difficult

Using hand over hand assistance where necessary

Take towel and dry affected hand then using assistance place towel in affected. Complete task bimanually if possible

To dry unaffected facilitate where necessary giving proprioceptive feedback.

To wash face will need some hand over hand assistance to bring affected hand up to face and do the activity bi manually.

Possible progression:

- Reduce the level of assistance
- Stop giving hand over hand when drying
- Progress to washing hair.

© RATULS study team 2015

2. Part-task activities:

Sitting on a perching stool/seat

W2.01 Move affected hand from lap to the table and back to lap

W2.02 Reach affected hand to sink and return to lap.

W2.03 Facilitate fingers of affected hand around soap then release.

As an alternative use a soaped flannel if the soap is too difficult.

W2.04 Reach and grasp soap with affected hand and lather with unaffected hand.

May require help with maintaining position

W2.05 Grasp soap with affected hand, lather with unaffected hand. Release soap and rinse.

W2.06 Grasp soap with affected hand, lather with unaffected hand. Release soap, rinse and take towel in unaffected hand to dry.

W2.07 Move lathered hands to appropriate body part and back and use unaffected hand to dry.

Assist with unaffected arm or therapist to facilitate if required.

GOAL ACHIEVED

Possible progression:

- Reduce the level of assistance
- Progress to washing hair
- Reduce the amount of facilitation

W3 – Cleaning Teeth

1. Whole-task activity:

Sitting on a perching stool/seat

NO GRASP

One hand at the elbow and another around the hand to facilitate grip.

1. With hand over hand assistance, facilitate picking up brush with affected hand.
2. Facilitate hand to mouth and facilitate brushing. Check position of hand and arm constantly.
3. Move hand/arm towards sink – facilitating where necessary- to rinse brush.
4. Release the brush from affected hand.
5. Repeat the process.

SOME GRASP

1. Take hold of the toothbrush in the affected hand.
   - May need some assistance to get correct position.
2. Lift up to mouth – may need some assistance at elbow/wrist to maintain position.
3. Begin to brush teeth – encourage brushing on both sides of the mouth especially if inattentive to affected side.
   - Will need various levels of hand over hand assistance throughout the task.
4. Rinse brush under the tap – giving hand over hand assistance at elbow/wrist where necessary.
   - Discourage over use at the shoulder to compensate.
5. Release the brush from affected hand.
6. W3.05b Repeat the process.

Possible progression:

- Reduce the level of assistance/facilitation.
- If grip improves from no grip – move to opposite chart.
- Try putting toothpaste on toothbrush if grip improves.

© RATULS study team 2015

2. Part-task activities:

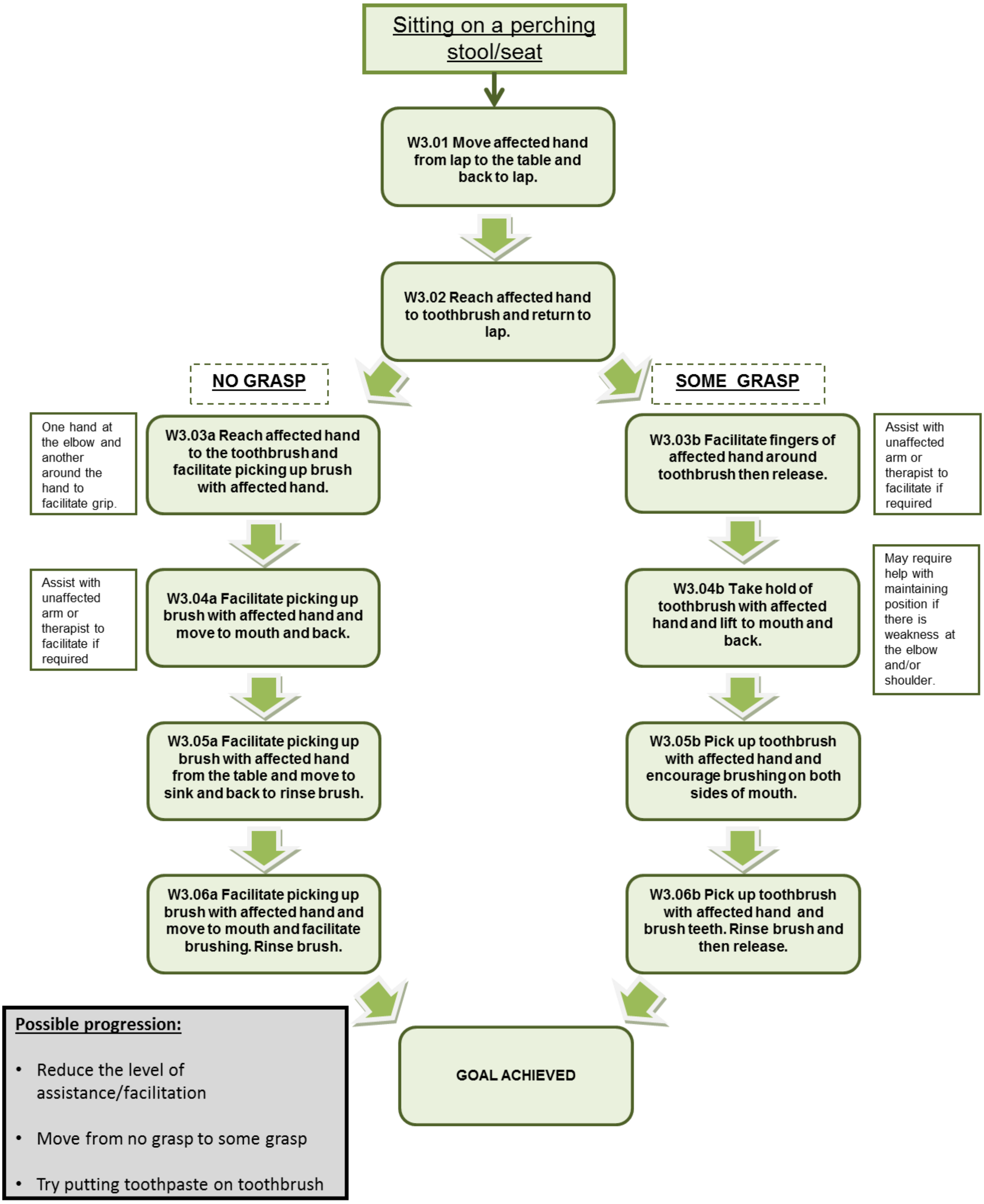

© RATULS study team 2015

W4 – Hand care

1. Whole-task activity:

Sit on a supportive chair with table in front at the correct height to place hands comfortably on the table.

Use perching stool or less support if wanting to work on sitting balance.

Place both arms on table making sure the elbows are supported.

May need some assistance/ supervision to get this position.

Using unaffected hand turn affected hand to face palm up. Stretch out the fingers and the thumb individually.

May need towel to place affected hand on.

Place hand cream onto palm and massage in to hand – making sure it's in between fingers as well and along each individual finger/thumb.

May need some supervision to maintain position in supination.

Turn hand over and repeat on dorsum of hand.

Turn hand back over and bend fingers to form a fist – taking care to include each finger. Then stretch out and take thumb across palm to base of little finger and back.

If there is activity in the hand – encourage finger flexion actively as far as possible and then to use unaffected hand to force the fist.

Repeat the process as necessary.

Possible progression:

- Maintain hand position without assistance.
- Include nail care- filing, polishing.
- Rubbing hands together to rub in cream.

© RATULS study team 2015

2. Part-task activities:

<u>Sit on a supportive chair with table in front at the correct height to place hands comfortably on the table.</u>

W4.01 Move both hands from lap to the table and back to lap.

W4.02 Use unaffected hand to turn affected hand palm up and return to original position.

W4.03 Use unaffected hand to turn affected hand palm up and facilitate finger extension.

W4.04 Facilitate finger extension with unaffected hand and place hand cream onto palm and massage into hand and fingers.

May require help with maintaining position

W4.05 Facilitate finger extension with unaffected hand and place hand cream onto dorsum of hand and massage into hand.

If there is activity in the hand encourage finger flexion actively as far as possible and then used unaffected hand to force fist

GOAL ACHIEVED

Possible progression:

- Maintain hand position without assistance
- Include nail care: filing/polishing
- Rubbing hands together to rub in cream.

© RATULS study team 2015

Dressing (D)

D1 – Closing a zip/ buttons

1. Whole-task activity:

Sitting on a perching stool/seat

This can be changed to more unstable surface (stool) to incorporate balance.

MODERATE GRIP

1. Use affected hand to stabilise - holding onto the bottom of the cardigan.
2. May need to facilitate the fingers around – and maintain it throughout the task.

GOOD GRIP

1. Use affected hand to pull up zip while stabilising with unaffected.
2. Encourage pincer grip with thumb and index finger to grasp the zip. May need support if there is poor sensation.
   May need help with securing the zip at the bottom.
3. Once grasped, pull zip up – may need support at the elbow and shoulder if there is instability around elbow and shoulder.
4. Throughout the task – may need facilitation at the wrist at certain points throughout the task.

Possible progression:

- Move from moderate to good
- Encourage patient to put zip together at start of the task.

© RATULS study team 2015

2. Part-task activities:

Sitting on a perching stool/seat

This can be changed to more unstable surface (stool) to incorporate balance.

D1.01 With cardigan on table move affected hand to cardigan and back from table.

Assist with unaffected arm or therapist to facilitate if required.

D1.02 With cardigan on table move affected hand to cardigan and encourage pincer grip with thumb and index finger to grasp the zip.

May need support if there is poor sensation.

D1.03 With cardigan on the table grasp the zip with affected hand and pull zip up with the unaffected hand.

May need help with securing the zip at the bottom.

Cardigan remains on table

D1.04a Facilitate fingers around the cardigan to stabilise and maintain whilst using unaffected hand to close zip/ buttons.

Patient wearing cardigan

D1.04b With the cardigan on patient move affected hand to bottom of cardigan and facilitate fingers around the cardigan to stabilise.

May need support at the elbow and shoulder if there is instability around elbow and shoulder.

D1.05b Facilitate fingers around the cardigan to stabilise and maintain whilst using unaffected hand to close zip/ buttons.

May need help with securing the zip at the bottom.

Possible progression:

- Move from cardigan on table to patient wearing cardigan (or vice versa)
- Encourage patient to put zip together at start of the task.

© RATULS study team 2015

D2 – Putting on a cardigan/ coat/ shirt

1. Whole-task activity:

Sitting on a supported surface

This can be changed to more unstable surface (stool) to incorporate balance

Always start with affected arm first

Lay garment across the lap with affected sleeve on the outside.

If limited function - to gather up sleeve and place it open in lap between the knees.

Place affected arm in the sleeve opening.

Give assistance/facilitation where required

Pull sleeve up arm so that the hand is through the sleeve – making sure it is past the elbow.

Continue to pull up and over the shoulder and as far around the back as possible.

Reach around with unaffected arm to put it through the sleeve.

Verbal and/or physical prompts may be required throughout this part of the task depending on the ability of the patient.

Repeat the process.

Possible progression:

- Use of time
- Less prompts –verbal /physical
- To carry out the task in standing
- Adjust for t-shirt or jumper.

© RATULS study team 2015

2. Part-task activities:

Sitting on a supported surface

This can be changed to more unstable surface (stool) to incorporate balance

Always start with affected arm first

D2.01 Move affected hand from lap to the table and back to lap.

Assist with unaffected arm or therapist to facilitate if required.

D2.02 Reach and grasp cardigan/ coat with unaffected hand and gather up sleeve of affected side.

Give assistance/facilitation where required

D2.03 Reach and grasp cardigan/coat with unaffected hand and lace affected arm in the sleeve opening.

Give assistance/facilitation where required

D2.04 Place affected arm in the sleeve opening so that the hand is through the sleeve and making sure the sleeve is above the elbow.

Verbal and/or physical prompts may be required throughout depending on the ability of the patient.

D2.05 Place affected arm in the sleeve opening and pull up over the shoulder.

D2.06 Place affected arm in the sleeve opening and pull up over the shoulder and around back.

D2.07 Place affected arm in the sleeve opening and pull up over the shoulder and around back placing unaffected arm in sleeve.

GOAL ACHIEVED

Possible progression:

- Use of time
- Less prompts –verbal /physical
- To carry out the task in standing.

© RATULS study team 2015

D3 – Putting on a mitten/ glove

1. Whole-task activity:

Sitting on a perching stool/seat

NO GRIP (mitten)

1. Place affected hand securely into lap.
2. Extend fingers and thumb.
3. Take glove in unaffected hand and put over extended fingers of affected.
   - May need some assistance - esp. if sensory problem.
4. Pull over thumb and enclosing all fingers then pull up over wrist making sure fingers are extended.
5. Take off glove and repeat the process.

SOME GRIP (glove)

1. Place affected hand into lap.
2. Place glove over extended fingers and thumb.
   - Assist with unaffected arm or therapist to facilitate if required.
3. Once over base of thumb – extend fingers.
4. Use unaffected hand to push down web of each individual finger and thumb.
5. Pull up glove securely.
6. Take off glove and repeat the process.

Possible progression:

- Move from mitten to glove
- Less supervision/assistance
- Try putting the glove on unaffected hand

© RATULS study team 2015

2. Part-task activities:

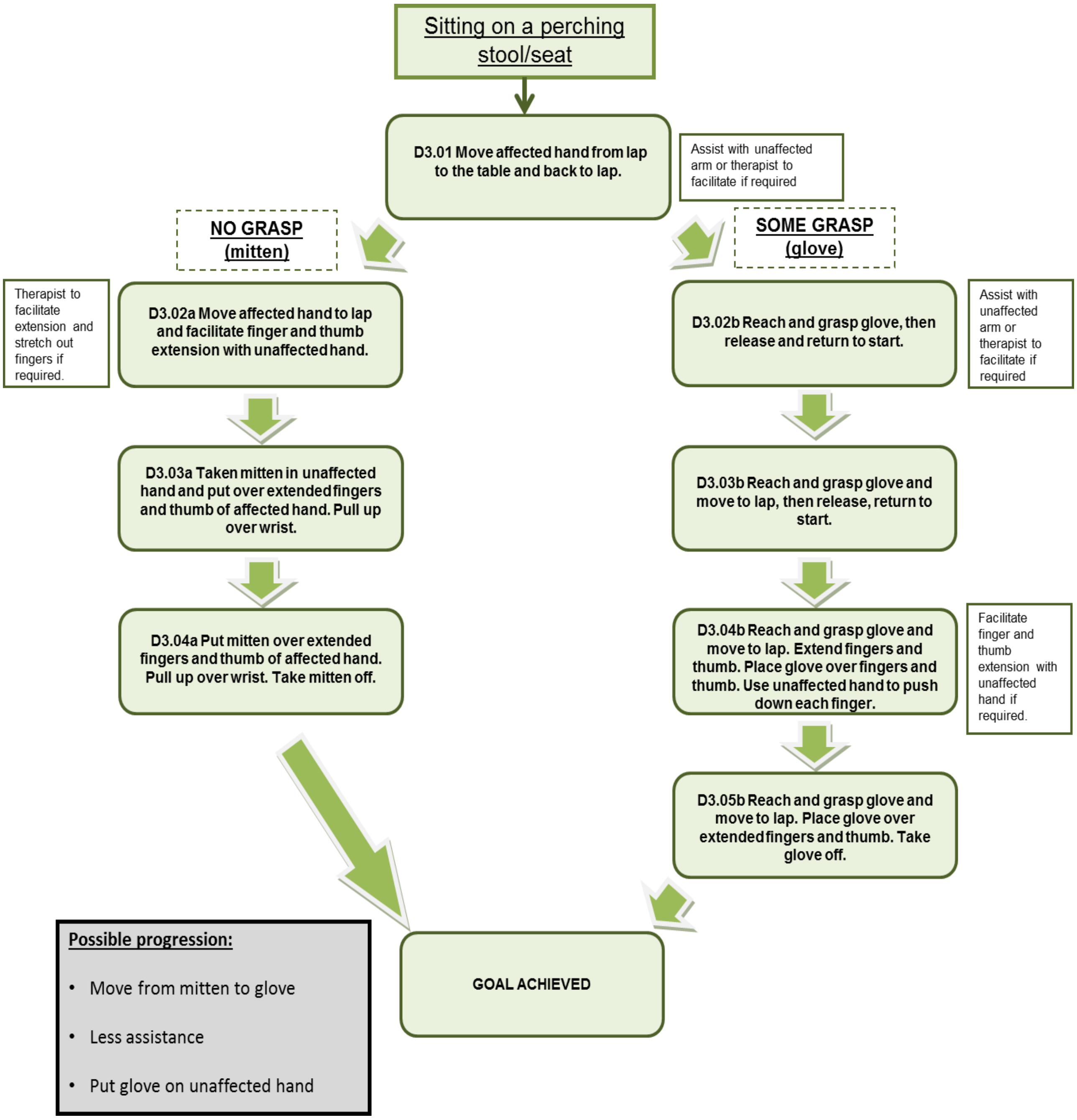

Possible progression:

- Move from mitten to glove
- Less assistance
- Put glove on unaffected hand

D4 – Putting on socks/ shoes

1. Whole-task activity:

NB: Only for participants with moderate to good grip and fairly good lower limb function.

Sitting securely with foot on a small stool or if possible cross leg over.

Increase/ decrease height of stool to make it easier or harder.

↓

Place sock over affected hand, gathered, in a lumbrical position over as far as the IP joints.

May need some assistance with this depending on activity throughout arm and hand.

↓

Lean forward and place sock over toes starting at little toe and slide hand out.

Depending on sitting balance may need some support leaning forward.

↓

Use both hands to pull sock up around heel – making sure the affected hand completes the task bi-manually.

↓

Take off sock and repeat the process.

Possible progression:

- Lower the height of the stool
- Try with leg crossed over
- Use tighter socks

© RATULS study team 2015

2. Part-task activities:

NB: Only for participants with moderate to good grip and fairly good lower limb function.

Sitting securely with foot on a small stool or if possible cross leg over

D4.01 Reach affected hand to foot, touch and return.

Assist with unaffected arm or therapist to facilitate if required.

D4.02 With unaffected hand, place sock over affected hand, gathered, in a lumbrical position over as far as the IP joints

Therapist to facilitate if required.

D4.03 With unaffected hand, place sock over affected hand (as above), lean forward and reach to knee and back.

May require assistance for sitting balance and support leaning forward.

D4.0.4 With unaffected hand, place sock over affected hand (as above), lean forward and reach to foot and back.

D4.05 With unaffected hand, place sock over affected hand (as above), lean forward put sock over toes. Start at little toe and slide hand out.

D4.06 With unaffected hand, place sock over affected hand (as above), lean forward put sock over toes. Use both hands to pull socks up bimanually.

D4.07 With unaffected hand, place sock over affected hand (as above), lean forward put sock on and pull up. Remove sock.

GOAL ACHIEVED

Possible progression:

- Lower height of stool
- Try with legs crossed over
- Use tighter socks.

© RATULS study team 2015

D5 – Putting on spectacles

1. **Whole-task activity:**

NB: The participant must have some grip.

Sitting at a table with spectacles in front of you.

↓

Using a pincer grip take hold of the arms of the spectacles – bilaterally.

Depending on grip – may require a level of assistance here and to maintain the grip position on the arm of the spectacles.

↓

Lift spectacles up to your face and place the arms over your ears. Make sure that the movement is kept level throughout.

May require assistance throughout the movement at the elbow and wrist depending on activity throughout the arm.

↓

Take off spectacles – repeating the process in reverse.

↓

Repeat as required.

Possible progression:

- Reduce the amount of assistance needed at the grip and elbow.
- Do it with only the affected arm if it is the dominant one.

© RATULS study team 2015

2. Part-task activities:

Sitting on a perching stool/seat

D5.01 Move affected hand from lap to the table (to spectacles) and back to lap.

Assist with unaffected arm or therapist to facilitate if required

SOME GRIP

Assist with unaffected arm or therapist to facilitate if required

D5.02a Move affected hand to chest and back to table. Task to be completed without spectacles.

D5.03a Move affected hand to face and back to table. Task to be completed without spectacles.

D5.04a Extend fingers around spectacle arms and release.

Bimanual = both hands involved doing different things e.g. knife and fork.

D5.05a Lift spectacles to face (bimanual with affected leading) and back to table.

D5.06a Lift spectacles to face (bimanual with affected leading) and place arms of spectacles over ears.

GOOD GRIP

D5.02b Reach and grip the arms of the spectacles (bilaterally), then release and return hands to front of table.

Assist with unaffected arm or therapist to facilitate if required

D5.03b Reach and grip spectacles and move closer to self, then release. Return hands to the front of the table.

D5.04b Reach and pick up spectacles , move to chest. Place back on table and release.

D5.05b Reach and pick up spectacles, move to face. Place back on table and release.

May require assistance throughout movement at the elbow and wrist.

D5.06b Reach and pick up spectacles and place arms of spectacles over ears. Place back on table and release.

GOAL ACHIEVED

Possible progression:

- Reduce the amount of assistance needed
- Do it with only the affected arm if it is the dominant one

© RATULS study team 2015

Eating and Drinking (F)

F1 – Drinking from mug/ glass

1. Whole-task activity:

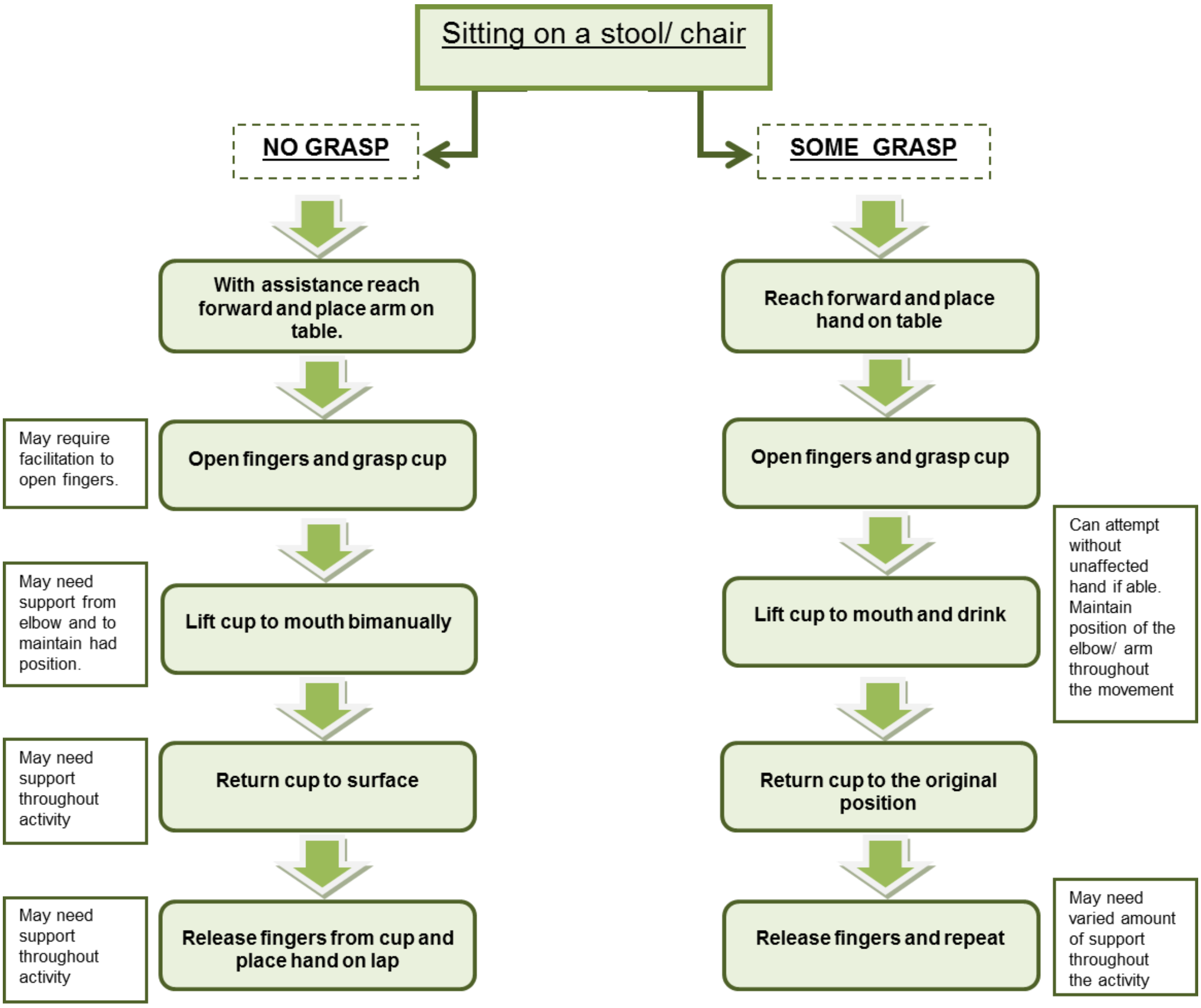

Possible progression:

- Alter size of cup/ mug
- Alter quantity of liquid
- Complete activity in standing
- Alter the amount of assistance

© RATULS study team 2015

2. Part-task activities:

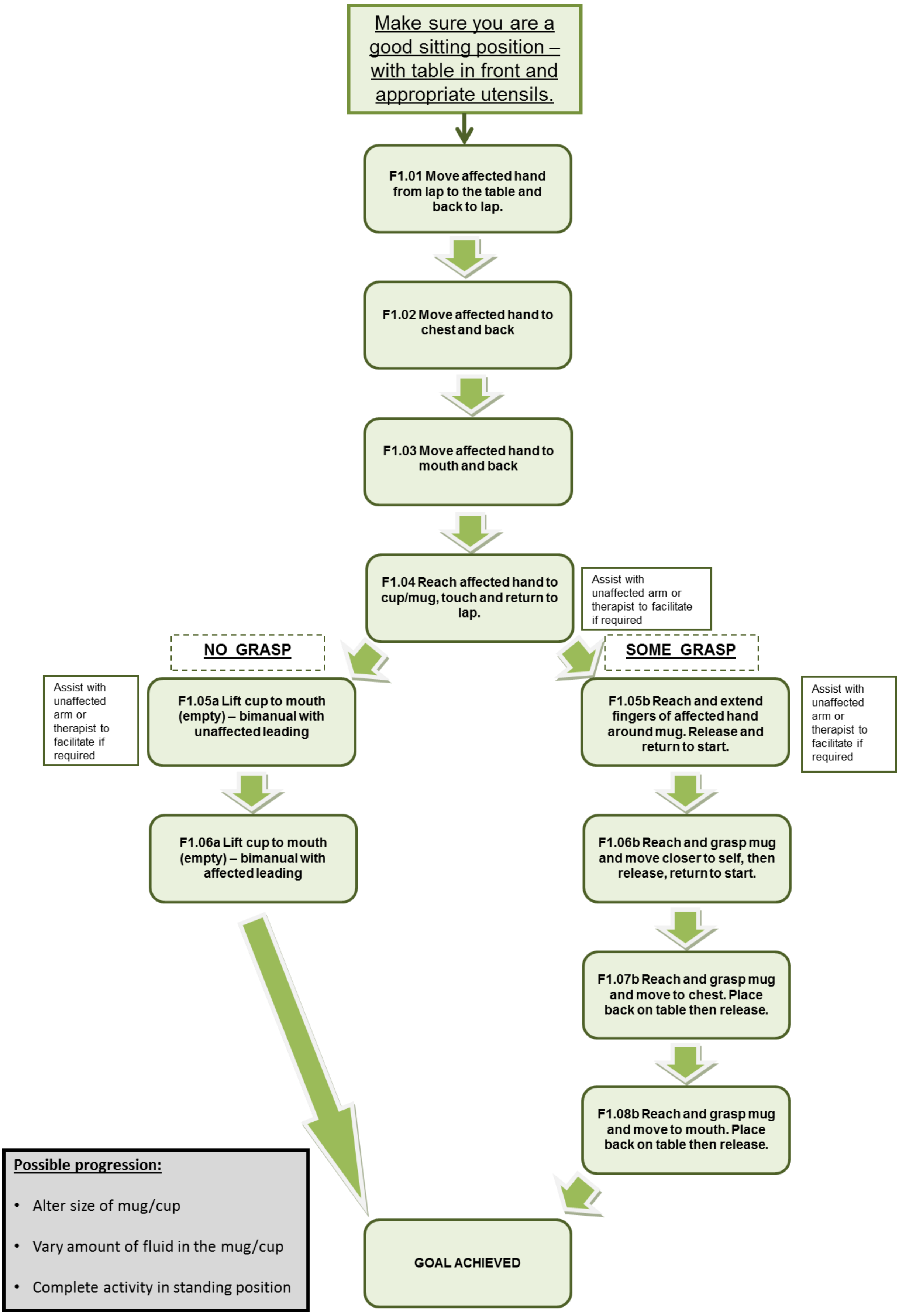

Possible progression:

- Alter size of mug/cup
- Vary amount of fluid in the mug/cup
- Complete activity in standing position

© RATULS study team 2015

F2 – Pouring from bottle/ jug/ kettle

1. Whole-task activity:

Make sure you are in a good sitting position – with table in front and appropriate utensils.

POOR GRIP

Place affected hand around the container to stabilise.

May need assistance to place hand around the container.

Maintain that position – holding it steady – while pouring with the unaffected arm.

May require help to maintain the position of the hand throughout the movement.

SOME GRIP

Reach forward with affected hand and grasp the bottle holding the container with unaffected hand.

May require help to open the fingers effectively to grasp the bottle.

Lift the bottle and pour – making sure that the shoulder stays down throughout the movement.

May require facilitation to keep hand around bottle and the assist in the correct elbow and shoulder movement.

Return bottle onto the table and let –go releasing fingers.

Need help to release fingers.

Possible progression:

- Move from poor grip to some grip
- Vary amount of fluid in the bottle
- Increase control required
- Reduce assistance/facilitation.

© RATULS study team 2015

2. Part-task activities:

Make sure you are in a good sitting position – with table in front and appropriate utensils.

F2.01 Move affected hand from lap to the table and back to lap.

F2.02 Reach affected hand to jug, touch and return to lap.

NO GRIP

May need facilitation to place hand on the jar and possibly to maintain it and some support at the elbow.

F2.03a Reach affected hand to the jug and facilitate fingers around jug to stabilise.

F2.04a Stabilise jug with affected hand and use unaffected hand to pour.

SOME GRIP

May require help with maintaining position if there is weakness at the elbow and/or shoulder.

F2.03b Stabilise jug with unaffected hand and reach affected hand to the handle of jug.

F2.04b Stabilise jug with unaffected hand and facilitate finger grip around the handle of jug – attempt to lift.

Watch movement at the wrist and assist where required.

F2.05b Stabilise jug with unaffected hand and grip handle of jug with affected hand. Lift and pour then return jug to table.

GOAL ACHIEVED

Possible progression:

- Move from poor grip to some grip
- Vary amount of fluid in the bottle
- Increase control required
- Reduce assistance/facilitation.

© RATULS study team 2015

F3 – Removing lids/ open containers

1. Whole-task activity:

Make sure you are in a good sitting position – with table in front and appropriate utensils.

NO GRIP

Will need facilitation to place hand on the jar and possibly to maintain it and some support at the elbow. Keep elbow on the table and maintain the position throughout the movement

- **Use affected hand to stabilise the jar while removing the lid with the unaffected hand.**
- **Release fingers from the jar – repeat process.**

SOME GRIP

- **Stabilise jar with unaffected hand.**
- **Place affected hand on top of lid and turn as able- keeping the elbow down.**
- **Remove lid from jar, open fingers and release lid onto table.**
- **Repeat the process.**

Check that the shoulder remains relaxed and not raised during the task. Watch movement at the wrist and assist where required. May require help with maintaining position if there is weakness at the elbow and/or shoulder.

Possible progression:

- Progress from no grip to some grip
- Tighten the lid
- Change size of jar

© RATULS study team 2015

2. Part-task activities:

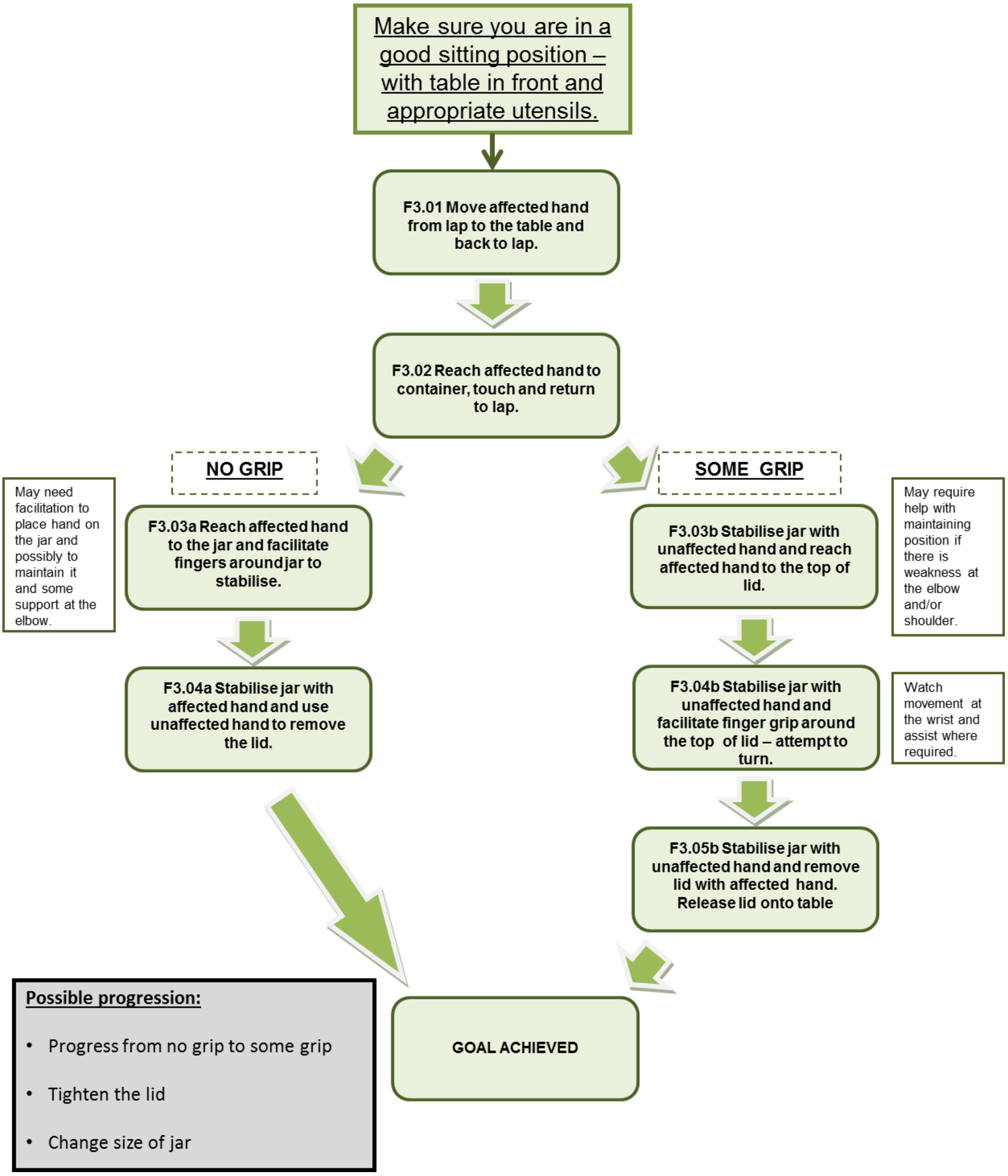

Possible progression:

- Progress from no grip to some grip
- Tighten the lid
- Change size of jar

© RATULS study team 2015

PC1 – Brushing hair

1. Whole-task activity:

Sitting on a supported surface

↓

Pick up brush from supporting surface.

May need some hand over hand assistance at the elbow and /or hand to keep the fingers around the brush.

↓

Lift to head – making sure arm is supported at the elbow and/or shoulder where required.

↓

Brush /comb hair – moving front to back as able- Keeping hand around the brush and making sure there is no shoulder pain.

May need varied amount of support – depending on the positioning of the elbow and shoulder and the ability to sustain the grip.

↓

Repeat the process

Possible progression:

- Reduce the amount of assistance required
- Change from brush-comb
- If high level try with hairdryer/tongs and do bimanually.

© RATULS study team 2015

2. Part-task activities:

<u>Sitting on a supported surface</u>

↓

PC1.01 Move affected hand from lap to the table and back to lap.

↓

PC1.02 Reach affected hand to brush and back to lap

↓

PC1.03 Use unaffected hand to facilitate fingers of affected hand to grasp brush and release.

↓

PC1.04 Grasp brush with affected hand and lift to chest and back to table. Release brush.

↓

PC1.05 Grasp brush with affected hand and lift to head and back to table. Release brush.

May require help with maintaining position

↓

PC1.06 Grasp brush with affected hand and lift to hair. Brush hair moving front to back with support from unaffected hand if needed.

↓

GOAL ACHIEVED

<u>Possible progression:</u>

- Reduce the amount of assistance required
- Change from brush to comb
- If high level try with hairdryer/ tongs and do bimanually.

© RATULS study team 2015

FM1 – Opening doors with affected hand

1. Whole-task activity:

In standing or in sitting with door handle board

↓

Reach forward from the shoulder – take hold of the handle.

May need some facilitation at shoulder and elbow

↓

Grasp the handle as able and turn or press down depending on handle.

May need help to stabilise the upper arm and shoulder and possibly to maintain the grip on the handle.

↓

Bend the elbow and pull handle/door towards you – stepping back where required.

If in standing ensure that patient is in a suitable position for safely opening/ closing the door

↓

Release the handle. The repeat the process in reverse – closing the door.

May require assistance throughout the movement.

Possible progression:

- Reduce the amount of assistance
- Try different handles of differing shapes.
- Progress to standing

© RATULS study team 2015

2. Part-task activities:

In standing or in sitting with door handle board

FM1.01 Move affected hand to the door handle and back.

May require some facilitation at shoulder and elbow.

FM1.02 Reach affected hand to door handle and facilitate fingers around handle.

FM1.03 Grasp the handle with affected hand and push/ turn the handle. Release the door handle.

May require help with maintaining position

FM1.04 Grasp the handle with affected hand and push/ turn the handle. Bend the elbow and pull door towards.

If in standing ensure that patient is in a suitable position for safely opening/ closing the door

FM1.05 Repeat above in reverse to close door.

GOAL ACHIEVED

Possible progression:

- Reduce the amount of assistance required
- Try different handle shapes
- Progress to standing

© RATULS study team 2015

FM2 – Sit-to-stand using both hands

1. Whole-task activity:

NB participant must be able to stand with assistance from 1

Sitting in a comfortable chair with arms and not too low making sure knees and hips are as close to 90 degrees as possible.

↓

Place hands on the arms and lean forward putting some weight through both arms.

Assistance may be required for positioning of hand on the arm and possibly for maintaining its position.

↓

Stand up – Pushing up through both arms and releasing when appropriate.

May need hands –on assistance throughout the whole movement to maintain hand position.

↓

From standing – reach back with both arms – elbow extended – hold onto arms and sit down.

↓

Repeat as many times as able/required.

Possible progression:

- Reduce the level of assistance.
- Different chair height
- Only use affected arm

Additional instructions for moving from sit to stand:

Please instruct the participant as follows:

1. place both hands (or, if not possible, the less-affected hand only) on the arm rests, or on the seat of the chair
2. shuffle bottom forward to the edge of the chair
3. move both feet back a little, keeping feet flat on the floor - and hip-width apart
4. in one movement, stand up as follows: keep looking straight ahead, lean forward from the hips and push down with your hands.
5. remain standing for a moment.

In people with an ankle foot orthosis (AFO) the affected foot is only to be moved back as far as is comfortable.

Additional instructions for moving from stand to sit:

Please instruct the participant as follows:

1. with the chair behind you, shuffle back until you can feel the seat of the chair against the back of your legs
2. check to see if there are arm rests
3. bending from the hips, reach for the arm rests, or for the seat of the chair.
4. gently lower yourself down.

© RATULS study team 2015

2. Part-task activities:

NB participant must be able to stand with assistance from 1

Sitting in a comfortable chair with arms and not too low making sure knees and hips are as close to 90 degrees as possible

FM2.01 Move hands to the chair arms and back to lap.

Assistance may be required for positioning of hand on arm and possibly for maintaining position.

FM2.02 Push through both arms to stand up. Release arms of chair when appropriate.

May need hands-on assistance throughout the whole movement to maintain hand position.

FM2.03 From standing reach back with both hand to chair arms and return to sides.

May require help with maintaining position

FM2.04 From standing reach back with both hand to chair arms, hold onto arms and sit down.

GOAL ACHIEVED

Possible progression:

- Reduce the level of assistance
- Different chair height
- Only use affected arm

5. Enhanced upper limb therapy documents

This Enhanced upper limb therapy 3: Warm-up stretches, goal choices and activity flowcharts manual is part of a series of documents developed to describe the RATULS enhanced upper limb therapy programme. The other documents in this series are:

- Enhanced upper limb therapy 1: Programme overview
- Enhanced upper limb therapy 2: How to deliver the programme

6. RATULS co-ordinating centre

For further information please contact the RATULS co-ordinating team:

Stroke Research Group

3-4 Claremont Terrace

Newcastle University

Newcastle Upon Tyne

NE2 4AE

Email: ratuls@ncl.ac.uk

Phone: 0191 2086779

Or refer to the RATULS website: http://research.ncl.ac.uk/ratuls/

© RATULS study team 2015

This enhanced upper limb therapy manual has been produced by Dr Helen Bosomworth with input from Miss Lianne Brkic, Mrs Lydia Aird, Dr Lisa Shaw and Professor Frederike van Wijck.

© RATULS study team 2015:
Helen Rodgers, Newcastle University
Lydia Aird, Northumbria Healthcare NHS Foundation Trust
Sreeman Andole, Barking, Havering & Redbridge University Hospitals NHS Trust
David Cohen, London North West Hospitals NHS Trust
Jesse Dawson, University of Glasgow
Janet Eyre, Newcastle University
Tracy Finch, Newcastle University
Gary Ford, Newcastle University
Steven Hogg, lay member
Denise Howel, Newcastle University
Niall Hughes, NHS Greater Glasgow and Clyde
Hermano Igo Krebs, Massachusetts Institute of Technology
Chris Price, Northumbria Healthcare NHS Foundation Trust
Lynn Rochester, Newcastle University
Lisa Shaw, Newcastle University
Laura Ternent, Newcastle University
Duncan Turner, University of East London
Luke Vale, Newcastle University
Frederike van Wijck, Glasgow Caledonian University
Scott Wilkes, University of Sunderland

National Institute for Health Research

Newcastle University

Robot Assisted Training for the Upper Limb after Stroke

RATULS

Enhanced upper limb therapy:

Participant goal and therapy record

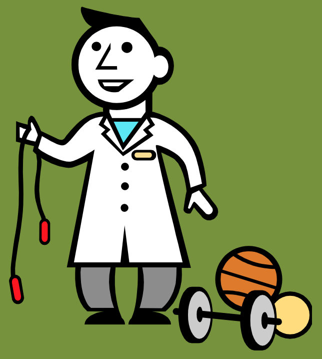

Participant ID

Study centre number

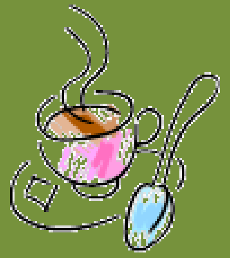

© RATULS study team 2015

Further information

Enhanced upper limb therapy documents

This Enhanced upper limb therapy: Participant goal and therapy record is designed to document the therapy received as part of the RATULS enhanced upper limb therapy programme. In order to deliver this programme please refer to the series of documents developed to describe the RATULS enhanced upper limb therapy programme. The documents in this series are:

- Enhanced upper limb therapy 1: Programme overview
- Enhanced upper limb therapy 2: How to deliver the programme
- Enhanced upper limb therapy 3: Warm-up stretches, goal choices and activity flowcharts.

RATULS co-ordinating centre

For further information please contact the RATULS co-ordinating team:

Stroke Research Group
3-4 Claremont Terrace
Newcastle University
Newcastle Upon Tyne
NE2 4AE
Email: ratuls@ncl.ac.uk
Phone: 0191 2086779

Or refer to the RATULS website: http://research.ncl.ac.uk/ratuls/

Programme overview

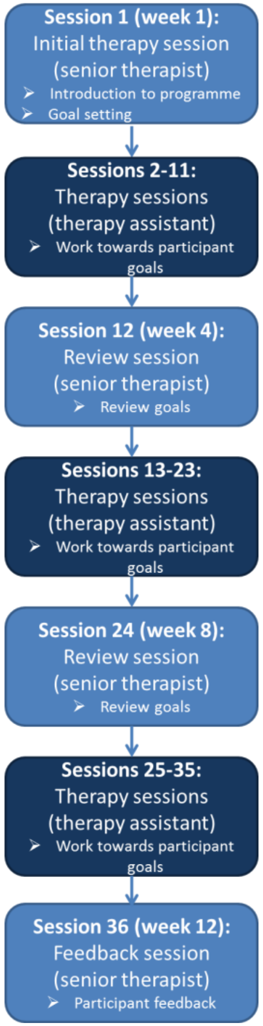

© RATULS study team 2015

Goal setting and review documentation

Participant ID: [ ] [ ] [ ] [ ] [ ]

Session 1<br>Week 1 Initial session	Goal 1				Goal 2				Goal 3				Goal 4			
	Whole (tick)	Part (tick)	Current Position	Part of task (? / ?)	Whole (tick)	Part (tick)	Current Position	Part of task (? / ?)	Whole (tick)	Part (tick)	Current Position	Part of task (? / ?)	Whole (tick)	Part (tick)	Current Position	Part of task (? / ?)
Session number:<br>Progress since week 1	Goal 1				Goal 2				Goal 3				Goal 4			
	Current Position		Part of task (? / ?)	Achieved (Y/N)	Current Position		Part of task (? / ?)	Achieved (Y/N)	Current Position		Part of task (? / ?)	Achieved (Y/N)	Current Position		Part of task (? / ?)	Achieved (Y/N)
Session number:<br>Week 4 Review session	Goal 1				Goal 2				Goal 3				Goal 4			
	Week 1 Goal 1 Achieved (Y/N)				Week 1 Goal 2 Achieved (Y/N)				Week 1 Goal 3 Achieved (Y/N)				Week 1 Goal 4 Achieved (Y/N)			
	Whole (tick)	Part (tick)	Current Position	Part of task (? / ?)	Whole (tick)	Part (tick)	Current Position	Part of task (? / ?)	Whole (tick)	Part (tick)	Current Position	Part of task (? / ?)	Whole (tick)	Part (tick)	Current Position	Part of task (? / ?)
Session number:<br>Progress since week 4	Goal 1				Goal 2				Goal 3				Goal 4			
	Current Position		Part of task (? / ?)	Achieved (Y/N)	Current Position		Part of task (? / ?)	Achieved (Y/N)	Current Position		Part of task (? / ?)	Achieved (Y/N)	Current Position		Part of task (? / ?)	Achieved (Y/N)
Session number:<br>Week 8 Review session	Goal 1				Goal 2				Goal 3				Goal 4			
	Week 4 Goal 1 Achieved (Y/N)				Week 4 Goal 2 Achieved (Y/N)				Week 4 Goal 3 Achieved (Y/N)				Week 4 Goal 4 Achieved (Y/N)			
	Whole (tick)	Part (tick)	Current Position	Part of task (? / ?)	Whole (tick)	Part (tick)	Current Position	Part of task (? / ?)	Whole (tick)	Part (tick)	Current Position	Part of task (? / ?)	Whole (tick)	Part (tick)	Current Position	Part of task (? / ?)
Session number:<br>Progress since week 8	Goal 1				Goal 2				Goal 3				Goal 4			
	Current Position		Part of task (? / ?)	Achieved (Y/N)	Current Position		Part of task (? / ?)	Achieved (Y/N)	Current Position		Part of task (? / ?)	Achieved (Y/N)	Current Position		Part of task (? / ?)	Achieved (Y/N)
Final session	Week 8 Goal 1 Achieved (Y/N)				Week 8 Goal 2 Achieved (Y/N)				Week 8 Goal 3 Achieved (Y/N)				Week 8 Goal 4 Achieved (Y/N)			

© RATULS study team 2015

Therapy record

Participant ID: | | | | | |

	Goal 1	Goal 2	Goal 3	Goal 4	Session notes
Session number:<br><br>Date:<br>___/____/20____<br><br>Session duration:<br>______mins					
	Repetitions	Repetitions	Repetitions	Repetitions	
	Therapist name:	___________________	Signature: __________	Duration of activity practice: ______mins	
	**Goal 1**	**Goal 2**	**Goal 3**	**Goal 4**	**Session notes**
Session number:<br><br>Date:<br>___/____/20____<br><br>Session duration:<br>______mins					
	Repetitions	Repetitions	Repetitions	Repetitions	
	Therapist name:	___________________	Signature: __________	Duration of activity practice: ______mins	
	**Goal 1**	**Goal 2**	**Goal 3**	**Goal 4**	**Session notes**
Session number:<br><br>Date:<br>___/____/20____<br><br>Session duration:<br>______mins					
	Repetitions	Repetitions	Repetitions	Repetitions	
	Therapist name:	___________________	Signature: __________	Duration of activity practice: ______mins	
	**Goal 1**	**Goal 2**	**Goal 3**	**Goal 4**	**Session notes**
Session number:<br><br>Date:<br>___/____/20____<br><br>Session duration:<br>______mins					
	Repetitions	Repetitions	Repetitions	Repetitions	
	Therapist name:	___________________	Signature: __________	Duration of activity practice: ______mins	

© RATULS study team 2015

Therapy record

Participant ID: ☐☐☐☐☐

Session number:<br>Date:<br>___/____/20____<br>Session duration:<br>______mins	Goal 1	Goal 2	Goal 3	Goal 4	Session notes
	Repetitions	Repetitions	Repetitions	Repetitions	
	Therapist name:	________________	Signature: __________	Duration of activity practice: ______mins	

Session number:<br>Date:<br>___/____/20____<br>Session duration:<br>______mins	Goal 1	Goal 2	Goal 3	Goal 4	Session notes
	Repetitions	Repetitions	Repetitions	Repetitions	
	Therapist name:	________________	Signature: __________	Duration of activity practice: ______mins	

Session number:<br>Date:<br>___/____/20____<br>Session duration:<br>______mins	Goal 1	Goal 2	Goal 3	Goal 4	Session notes
	Repetitions	Repetitions	Repetitions	Repetitions	
	Therapist name:	________________	Signature: __________	Duration of activity practice: ______mins	

Session number:<br>Date:<br>___/____/20____<br>Session duration:<br>______mins	Goal 1	Goal 2	Goal 3	Goal 4	Session notes
	Repetitions	Repetitions	Repetitions	Repetitions	
	Therapist name:	________________	Signature: __________	Duration of activity practice: ______mins	

© RATULS study team 2015

Therapy record

Participant ID: | | | | | |

	Goal 1	Goal 2	Goal 3	Goal 4	Session notes
**Session number:**<br><br>**Date:**<br>___/____/20____<br><br>**Session duration:**<br>______mins					
	**Repetitions**	**Repetitions**	**Repetitions**	**Repetitions**	
	**Therapist name:** ___________________		**Signature:** __________	**Duration of activity practice:** ______mins	

	Goal 1	Goal 2	Goal 3	Goal 4	Session notes
**Session number:**<br><br>**Date:**<br>___/____/20____<br><br>**Session duration:**<br>______mins					
	**Repetitions**	**Repetitions**	**Repetitions**	**Repetitions**	
	**Therapist name:** ___________________		**Signature:** __________	**Duration of activity practice:** ______mins	

	Goal 1	Goal 2	Goal 3	Goal 4	Session notes
**Session number:**<br><br>**Date:**<br>___/____/20____<br><br>**Session duration:**<br>______mins					
	**Repetitions**	**Repetitions**	**Repetitions**	**Repetitions**	
	**Therapist name:** ___________________		**Signature:** __________	**Duration of activity practice:** ______mins	

	Goal 1	Goal 2	Goal 3	Goal 4	Session notes
**Session number:**<br><br>**Date:**<br>___/____/20____<br><br>**Session duration:**<br>______mins					
	**Repetitions**	**Repetitions**	**Repetitions**	**Repetitions**	
	**Therapist name:** ___________________		**Signature:** __________	**Duration of activity practice:** ______mins	

© RATULS study team 2015

Therapy record

Participant ID: ☐☐☐☐☐

	Goal 1	Goal 2	Goal 3	Goal 4	Session notes
Session number:<br>Date:<br>___/____/20____<br>Session duration:<br>______mins					
	Repetitions	Repetitions	Repetitions	Repetitions	
	Therapist name:	___________________	Signature: __________	Duration of activity practice: ______mins	
	Goal 1	Goal 2	Goal 3	Goal 4	Session notes
Session number:<br>Date:<br>___/____/20____<br>Session duration:<br>______mins					
	Repetitions	Repetitions	Repetitions	Repetitions	
	Therapist name:	___________________	Signature: __________	Duration of activity practice: ______mins	
	Goal 1	Goal 2	Goal 3	Goal 4	Session notes
Session number:<br>Date:<br>___/____/20____<br>Session duration:<br>______mins					
	Repetitions	Repetitions	Repetitions	Repetitions	
	Therapist name:	___________________	Signature: __________	Duration of activity practice: ______mins	
	Goal 1	Goal 2	Goal 3	Goal 4	Session notes
Session number:<br>Date:<br>___/____/20____<br>Session duration:<br>______mins					
	Repetitions	Repetitions	Repetitions	Repetitions	
	Therapist name:	___________________	Signature: __________	Duration of activity practice: ______mins	

© RATULS study team 2015

Therapy record

Participant ID: ☐☐☐☐☐

Session number:<br>Date:<br>___/____/20____<br>Session duration:<br>______mins	Goal 1	Goal 2	Goal 3	Goal 4	Session notes
	Repetitions	Repetitions	Repetitions	Repetitions	
	Therapist name: ____________________		Signature: __________	Duration of activity practice: ______mins	

Session number:<br>Date:<br>___/____/20____<br>Session duration:<br>______mins	Goal 1	Goal 2	Goal 3	Goal 4	Session notes
	Repetitions	Repetitions	Repetitions	Repetitions	
	Therapist name: ____________________		Signature: __________	Duration of activity practice: ______mins	

Session number:<br>Date:<br>___/____/20____<br>Session duration:<br>______mins	Goal 1	Goal 2	Goal 3	Goal 4	Session notes
	Repetitions	Repetitions	Repetitions	Repetitions	
	Therapist name: ____________________		Signature: __________	Duration of activity practice: ______mins	

Session number:<br>Date:<br>___/____/20____<br>Session duration:<br>______mins	Goal 1	Goal 2	Goal 3	Goal 4	Session notes
	Repetitions	Repetitions	Repetitions	Repetitions	
	Therapist name: ____________________		Signature: __________	Duration of activity practice: ______mins	

© RATULS study team 2015

Therapy record

Participant ID: ☐☐☐☐☐

Session number:<br><br>Date:<br>___/____/20____<br><br>Session duration:<br>______mins	Goal 1	Goal 2	Goal 3	Goal 4	Session notes
	Repetitions	Repetitions	Repetitions	Repetitions	
	Therapist name: ________________		Signature: __________	Duration of activity practice: ______mins	

Session number:<br><br>Date:<br>___/____/20____<br><br>Session duration:<br>______mins	Goal 1	Goal 2	Goal 3	Goal 4	Session notes
	Repetitions	Repetitions	Repetitions	Repetitions	
	Therapist name: ________________		Signature: __________	Duration of activity practice: ______mins	

Session number:<br><br>Date:<br>___/____/20____<br><br>Session duration:<br>______mins	Goal 1	Goal 2	Goal 3	Goal 4	Session notes
	Repetitions	Repetitions	Repetitions	Repetitions	
	Therapist name: ________________		Signature: __________	Duration of activity practice: ______mins	

Session number:<br><br>Date:<br>___/____/20____<br><br>Session duration:<br>______mins	Goal 1	Goal 2	Goal 3	Goal 4	Session notes
	Repetitions	Repetitions	Repetitions	Repetitions	
	Therapist name: ________________		Signature: __________	Duration of activity practice: ______mins	

© RATULS study team 2015

Therapy record

Participant ID: ☐☐☐☐☐

	Goal 1	Goal 2	Goal 3	Goal 4	Session notes
Session number:<br><br>Date:<br>___/____/20____<br><br>Session duration:<br>______mins					
	Repetitions	Repetitions	Repetitions	Repetitions	
	Therapist name:	___________________	Signature: __________	Duration of activity practice: ______mins	

	Goal 1	Goal 2	Goal 3	Goal 4	Session notes
Session number:<br><br>Date:<br>___/____/20____<br><br>Session duration:<br>______mins					
	Repetitions	Repetitions	Repetitions	Repetitions	
	Therapist name:	___________________	Signature: __________	Duration of activity practice: ______mins	

	Goal 1	Goal 2	Goal 3	Goal 4	Session notes
Session number:<br><br>Date:<br>___/____/20____<br><br>Session duration:<br>______mins					
	Repetitions	Repetitions	Repetitions	Repetitions	
	Therapist name:	___________________	Signature: __________	Duration of activity practice: ______mins	

	Goal 1	Goal 2	Goal 3	Goal 4	Session notes
Session number:<br><br>Date:<br>___/____/20____<br><br>Session duration:<br>______mins					
	Repetitions	Repetitions	Repetitions	Repetitions	
	Therapist name:	___________________	Signature: __________	Duration of activity practice: ______mins	

© RATULS study team 2015

Therapy record

Participant ID: | | | | | |

Session	Goal 1	Goal 2	Goal 3	Goal 4	Session notes
Session number:<br>Date:<br>___/____/20____<br>Session duration:<br>______mins					
	Repetitions	Repetitions	Repetitions	Repetitions	
	Therapist name:	__________________	Signature: __________	Duration of activity practice: ______mins	

Session	Goal 1	Goal 2	Goal 3	Goal 4	Session notes
Session number:<br>Date:<br>___/____/20____<br>Session duration:<br>______mins					
	Repetitions	Repetitions	Repetitions	Repetitions	
	Therapist name:	__________________	Signature: __________	Duration of activity practice: ______mins	

Session	Goal 1	Goal 2	Goal 3	Goal 4	Session notes
Session number:<br>Date:<br>___/____/20____<br>Session duration:<br>______mins					
	Repetitions	Repetitions	Repetitions	Repetitions	
	Therapist name:	__________________	Signature: __________	Duration of activity practice: ______mins	

Session	Goal 1	Goal 2	Goal 3	Goal 4	Session notes
Session number:<br>Date:<br>___/____/20____<br>Session duration:<br>______mins					
	Repetitions	Repetitions	Repetitions	Repetitions	
	Therapist name:	__________________	Signature: __________	Duration of activity practice: ______mins	

© RATULS study team 2015

Therapy record

Participant ID: | | | | | |

	Goal 1	Goal 2	Goal 3	Goal 4	Session notes
Session number:					
Date: ___/____/20____	Repetitions	Repetitions	Repetitions	Repetitions	
Session duration: ______mins					
	Therapist name: ___________________		Signature: __________	Duration of activity practice: ______mins	

	Goal 1	Goal 2	Goal 3	Goal 4	Session notes
Session number:					
Date: ___/____/20____	Repetitions	Repetitions	Repetitions	Repetitions	
Session duration: ______mins					
	Therapist name: ___________________		Signature: __________	Duration of activity practice: ______mins	

	Goal 1	Goal 2	Goal 3	Goal 4	Session notes
Session number:					
Date: ___/____/20____	Repetitions	Repetitions	Repetitions	Repetitions	
Session duration: ______mins					
	Therapist name: ___________________		Signature: __________	Duration of activity practice: ______mins	

	Goal 1	Goal 2	Goal 3	Goal 4	Session notes
Session number:					
Date: ___/____/20____	Repetitions	Repetitions	Repetitions	Repetitions	
Session duration: ______mins					
	Therapist name: ___________________		Signature: __________	Duration of activity practice: ______mins	

© RATULS study team 2015

Final session feedback and advice

Participant ID: | | | | | |

Feedback given to participant about progress over the programme

Further advice given regarding maintaining upper limb function (e.g home exercises)

© RATULS study team 2015

Session notes continued

Participant ID: | | | | | |

Session notes
Session number:

Session notes
Session number:

Session notes
Session number:

Session notes
Session number:

Session notes
Session number:

Session notes
Session number:

© RATULS study team 2015

Session notes continued

Participant ID: | | | | | |

Session notes
Session number:

Session notes
Session number:

Session notes
Session number:

Session notes
Session number:

Session notes
Session number:

Session notes
Session number:

© RATULS study team 2015

Upper limb assessment

Upper limb assessment to be completed by senior therapist

Session number: _____
Side of body affected by current stroke: _______________

Prompts:

- Selective movement
- Passive range of movement
- Muscle tone
- Compensations
- Proprioception
- Associated reactions
- Pain

**Shoulder**		**Wrist**	
**Elbow**		**Hand**	

Inattention / other comments:

____

____

____

© RATULS study team 2015

Upper limb assessment

Upper limb assessment to be completed by senior therapist

Session number: _____
Side of body affected by current stroke: _______________

Prompts:

- Selective movement
- Passive range of movement
- Muscle tone
- Compensations
- Proprioception
- Associated reactions
- Pain

**Shoulder**		**Wrist**	
**Elbow**		**Hand**	

Inattention / other comments:
____

____

____

© RATULS study team 2015

Upper limb assessment

Upper limb assessment to be completed by senior therapist

Session number: _____
Side of body affected by current stroke: _______________

Prompts:

- Selective movement
- Passive range of movement
- Muscle tone
- Compensations
- Proprioception
- Associated reactions
- Pain

**Shoulder**		**Wrist**	
**Elbow**		**Hand**	

Inattention / other comments:

____

____

____

© RATULS study team 2015
